# Supplementary material for: Extravillous trophoblast cell lineage development is associated with active remodeling of the chromatin landscape
Source: Nat Commun. 2023 Aug 10;14:4826. doi: 10.1038/s41467-023-40424-5 (PMC10415281; doi:10.1038/s41467-023-40424-5)
Supplement: Supplementary file 1 — Supplementary Information [file 41467_2023_40424_MOESM1_ESM.pdf]

Supplementary Material

Extravillous trophoblast cell lineage development is associated with active remodeling of the chromatin landscape

Kaela M. Varberg[1,2,*,†], Esteban M. Dominguez[1,2,†], Boryana Koseva[3], Joseph M. Varberg[4], Ross P. McNally[1,2,¥], Ayelen Moreno-Irusta[1,2], Emily R. Wesley[3], Khursheed Iqbal[1,2], Warren A. Cheung[3], Carl Schwendinger-Schreck[3], Craig Smail[3], Hiroaki Okae[5], Takahiro Arima[5], Michael Lydic[6], Kristin Holoch[6], Courtney Marsh[1,6], Michael J. Soares[1,2,6,7,*], and Elin Grundberg[1,2,3,*]

[1]Institute for Reproductive and Developmental Sciences, University of Kansas Medical Center, Kansas City, KS, USA 66160
[2]Department of Pathology & Laboratory Medicine, University of Kansas Medical Center, Kansas City, KS, USA 66160
[3]Genomic Medicine Center, Children's Mercy Research Institute, Children's Mercy Kansas City, Kansas City, MO, USA 64108
[4]Stowers Institute for Medical Research, Kansas City, MO, USA 64110
[5]Department of Informative Genetics, Environment and Genome Research Center, Tohoku University Graduate School of Medicine, Sendai 980-8575, Japan
[6]Department of Obstetrics and Gynecology, University of Kansas Medical Center, Kansas City, KS, USA 66160
[7]Center for Perinatal Research, Children's Mercy Research Institute, Children's Mercy, Kansas City, MO, USA 64108

†Co-first authors.

***Correspondence:** Dr. Kaela M. Varberg or Michael J. Soares, The University of Kansas Medical Center, Department of Pathology and Laboratory Medicine**,** 3901 Rainbow Blvd, MS3050, Kansas City, KS, USA 66160, Phone: 913-588-5774, Fax: 913-588-7180, Email: kvarberg@kumc.edu or msoares@kumc.edu; or Dr. Elin Grundberg, Genomic Medicine Center, Children's Mercy Research Institute, Children's Mercy, Kansas City, MO, USA 64108, Phone: 816-731-7084, Email: egrundberg@cmh.edu

This PDF file includes:

Supplementary Figures 1-25
Supplementary Tables 1-4
Supplementary References

Other supplementary materials for this manuscript include the following:

Supplementary Data 1-17 (separate excel file)
Source Data file (separate excel file)

Table of Contents

Supplementary Figure 1 .... Page 3
Supplementary Figure 2 .... Page 4
Supplementary Figure 3 .... Page 5
Supplementary Figure 4 .... Page 7
Supplementary Figure 5 .... Page 8
Supplementary Figure 6 .... Page 10
Supplementary Figure 7 .... Page 12
Supplementary Figure 8 .... Page 13
Supplementary Figure 9 .... Page 14
Supplementary Figure 10 .... Page 16
Supplementary Figure 11 .... Page 18
Supplementary Figure 12 .... Page 20
Supplementary Figure 13 .... Page 22
Supplementary Figure 14 .... Page 24
Supplementary Figure 15 .... Page 26
Supplementary Figure 16 .... Page 27
Supplementary Figure 17 .... Page 28
Supplementary Figure 18 .... Page 29
Supplementary Figure 19 .... Page 30
Supplementary Figure 20 .... Page 31
Supplementary Figure 21 .... Page 32
Supplementary Figure 22 .... Page 34
Supplementary Figure 23 .... Page 35
Supplementary Figure 24 .... Page 36
Supplementary Figure 25 .... Page 37
Supplementary Table 1........ Page 38
Supplementary Table 2........ Page 39
Supplementary Table 3........ Page 40
Supplementary Table 4........ Page 41
References .... Page 42

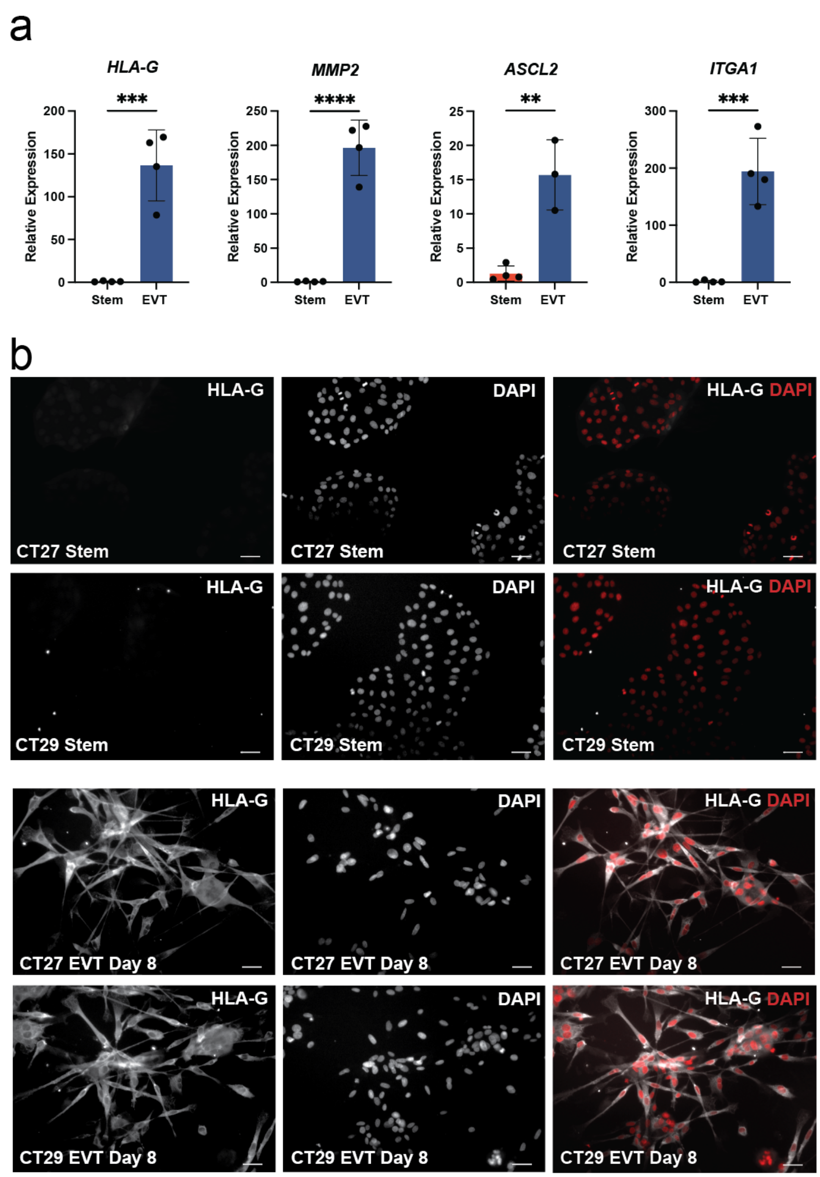

Supplementary Figure 1. Expression of EVT cell state-specific markers including HLA-G. **a)** Relative expression of *HLA-G* (p=0.0006), *MMP2* (p<0.0001), *ASCL2* (p=0.0025), and *ITGA1* (p=0.0006) measured by RT-qPCR in stem state and following EVT cell differentiation. Data were analyzed by unpaired t-test and are presented as mean values ± standard deviation (SD; n=4 biologically independent replicates per group except n=3 for *ASCL2* in EVT). **b)** EVT cell differentiation is accompanied by cell elongation and upregulation of HLA-G (white) in CT27 and CT29 cell lines. DAPI stains cell nuclei (white or red as labeled). Scale bars represent 100 μm. Source data are provided as a Source Data file.

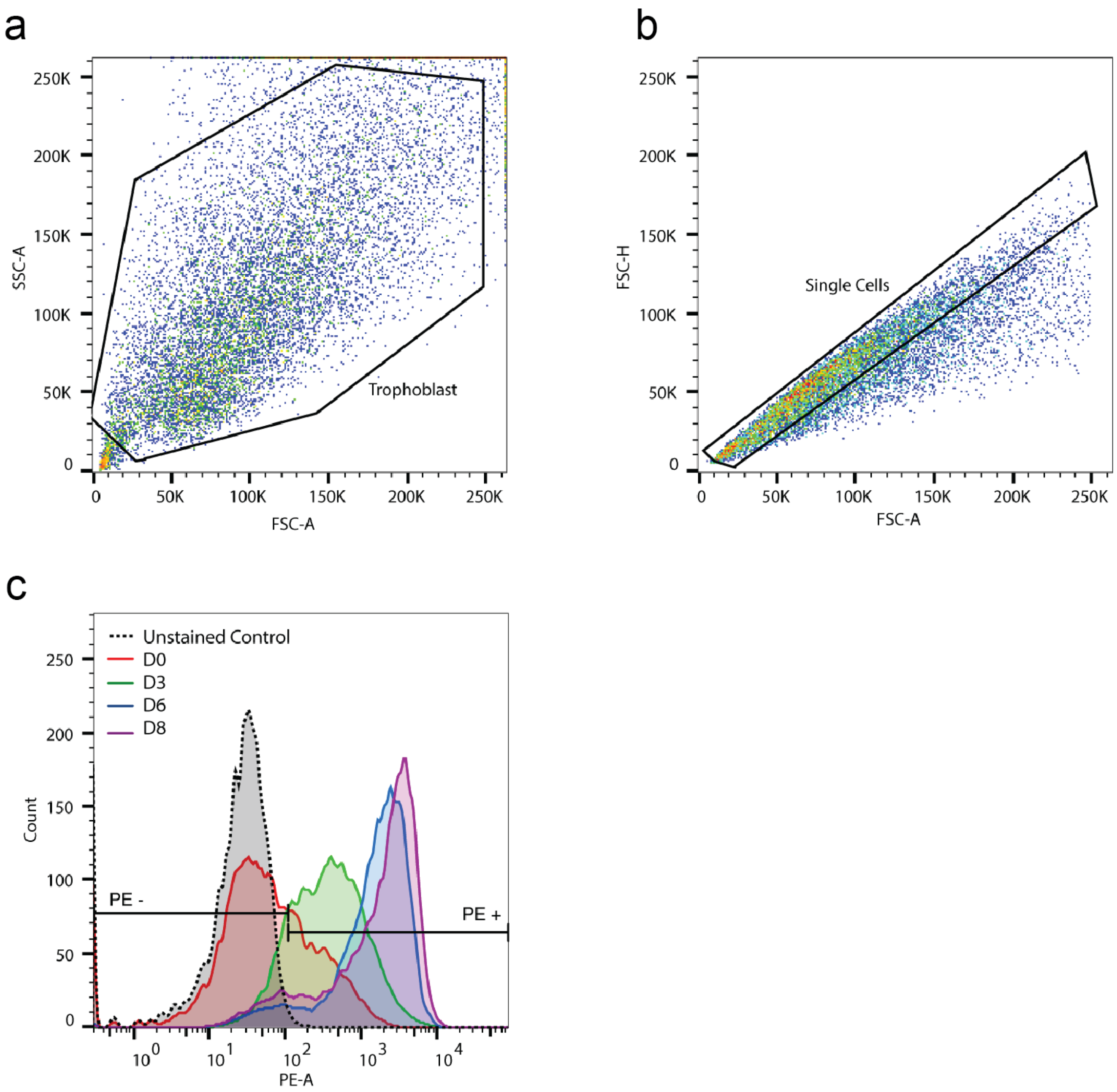

Supplementary Figure 2. Gating strategy for flow cytometric measurements of HLA-G. Representative plots depicting the gating strategy used to select for **a)** trophoblast cells (x-axis, forward scatter area, **FSC-A**; y-axis, side scatter area, SSC-A), followed by **b)** single cells (x-axis FSC-A; y-axis, forward scatter height, **FSC-H**), and then **c)** HLA-G positive cells (Phycoerythrin, **PE**+) depicted by black gates (x-axis, phycoerythrin area, PE-A; y-axis Count). Unstained control (black dotted line), stem state (D0 of EVT cell differentiation; red), day 3 (green), day 6 (blue), and day 8 (purple) of EVT cell differentiation are shown.

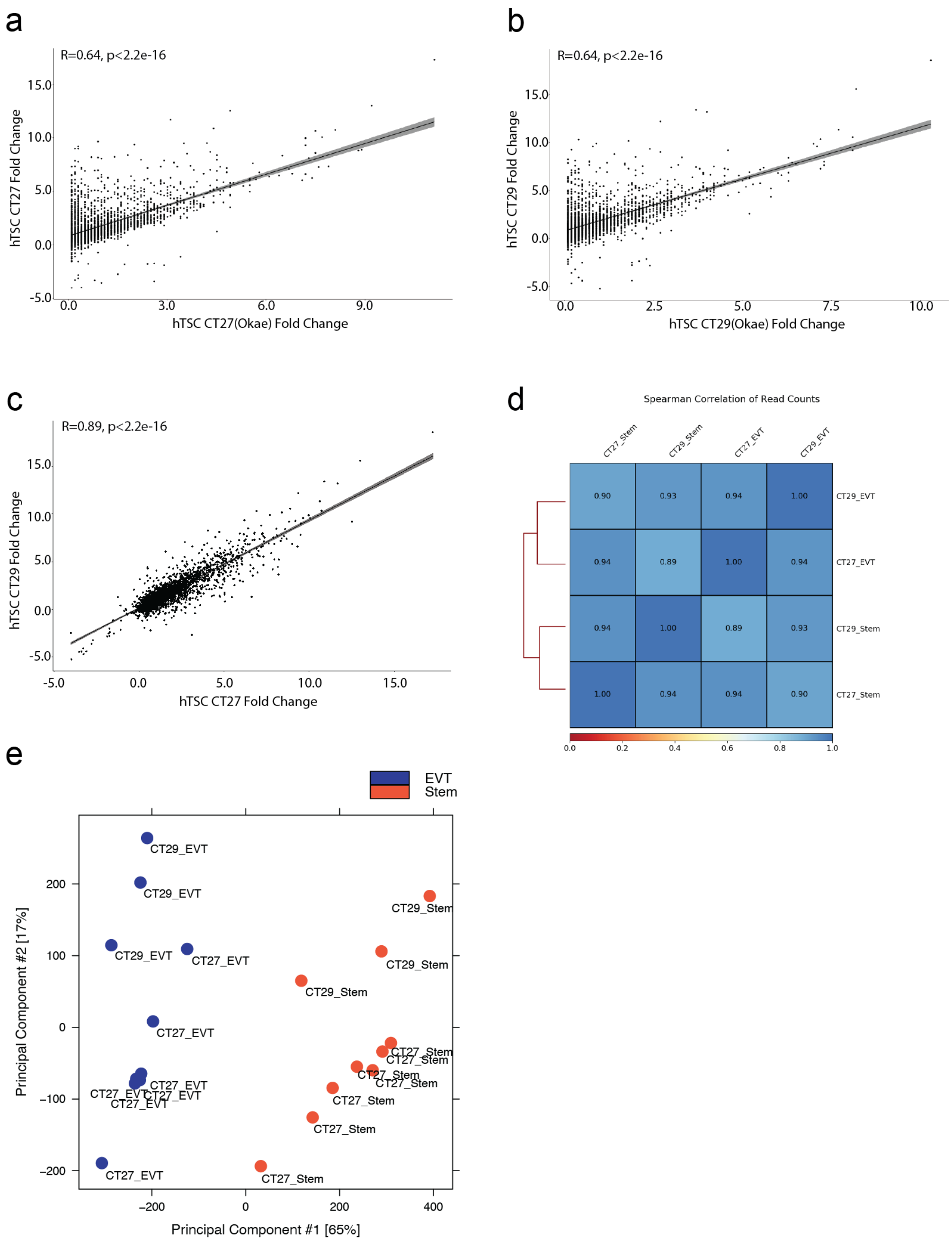

Supplementary Figure 3. Validation of transcriptomes across human TS cell donor lines and datasets. Scatter plots comparing log2 fold change from differential expression analysis of EVT cell transcriptomes relative to stem state transcriptomes in the CT27 (**a**) and CT29 (**b**) TS cell lines measured by RNA-Seq in this report (y-axis) and the Okae et al. (2018) report[1] (x-axis). Pearson correlations were calculated to estimate strength of the linear association ($p<2.2e-16$). **c)** Scatter plots comparing log2 fold change from differential expression analysis of

EVT cell transcriptomes relative to stem state transcriptomes for CT27 (x-axis) and CT29 (y-axis) TS cell lines generated from RNA-Seq and presented in this report. Pearson correlation was calculated to estimate strength of the linear association ($p<2.2e-16$). **d)** Spearman correlation heatmap using affinity (read count) data for EVT and Stem cell ATAC-Seq libraries for CT27 and CT29 TS cell lines. **e)** Principal component analysis (**PCA**) plot depicting EVT (blue) and Stem (red) cell data of counts per million mapped reads for independent ATAC-Seq libraries.

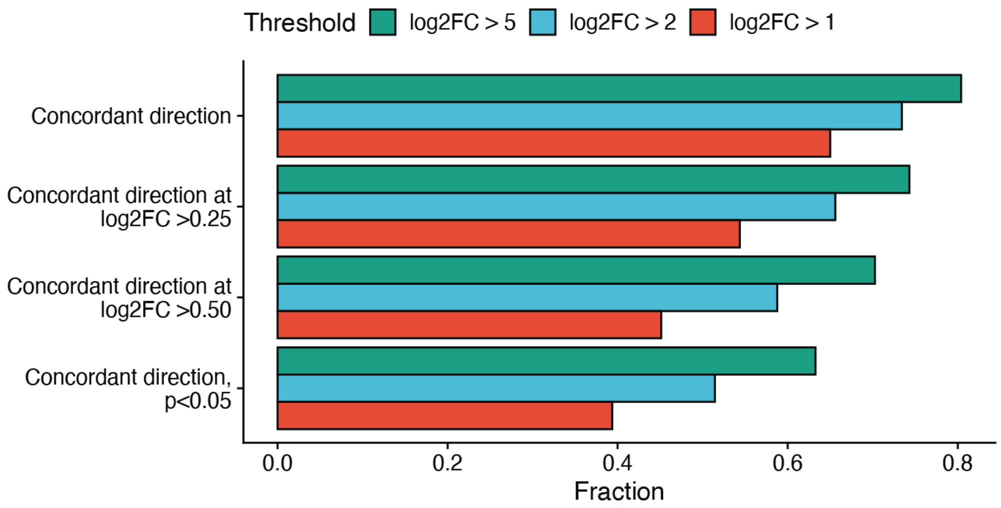

Supplementary Figure 4. Validation of gene expression patterns in EVT cells between *in vitro* and *in vivo* data sets. Bar graphs depicting concordance of differentially expressed genes detected in human TS cells (*in vitro*) compared to single-cell RNA expression data in first trimester placenta samples (*in vivo*). Concordance (Fraction, x-axis) is estimated for genes differently expressed in EVT cells compared to stem state cells at log2 fold-change > 5 (green bars), log2 fold-change > 2 (blue bars) and log2 fold-change > 1 (red bars) based on different threshold for differential expression in the *in vivo* data sets (y axis).

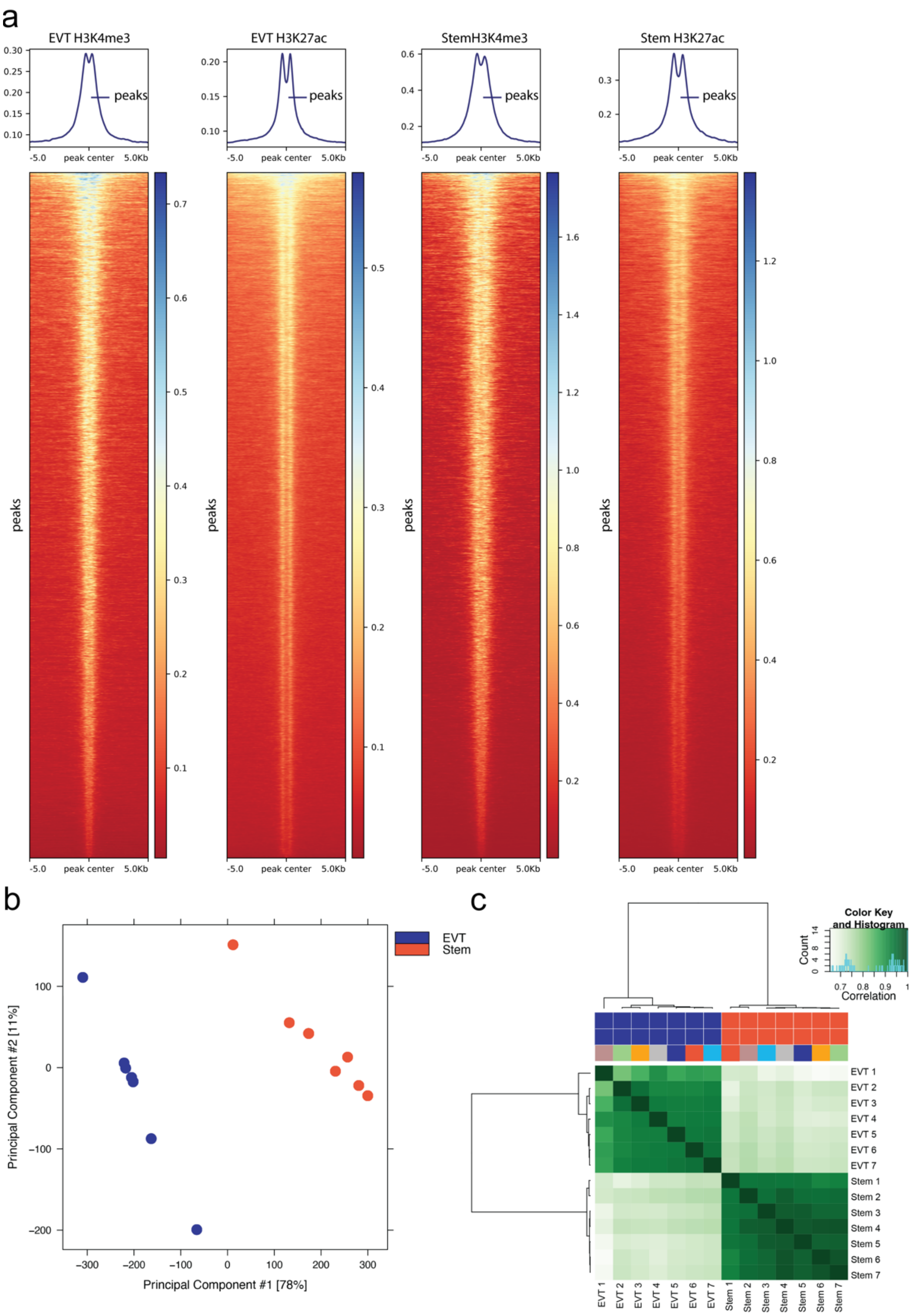

Supplementary Figure 5. Chromatin accessibility assayed by ATAC-Seq in stem and EVT cell-differentiated human TS cells a) Density plot and heatmap of coverages from EVT and Stem state ATAC-seq assays, respectively, centered around EVT H3K4me3 peaks (first, ranked

based on coverage in EVT ATAC-Seq un descending order), EVT H3K27ac peaks (second, ranked based on coverage in EVT ATAC-Seq un descending order), Stem state H3K4me3 peaks (third, ranked based on coverage in Stem state ATAC-Seq un descending order), and Stem state H3K27ac peaks (last, ranked based on coverage in Stem state ATAC-Seq un descending order). Each row represents one genomic region centered around (5kb) a peak and the depth of coverage is encoded in a colorimetric scale (red to blue corresponding low to high coverage). **b)** Principal component analysis (PCA) plot depicting EVT (blue) and Stem (orange) cell data of normalized read counts for independent ATAC-Seq libraries for all regions tested in the CT27 cell line. **c)** Correlation heatmap using affinity (read count) data for EVT (blue) and Stem (orange) cell ATAC-Seq libraries generated from the CT27cell line.

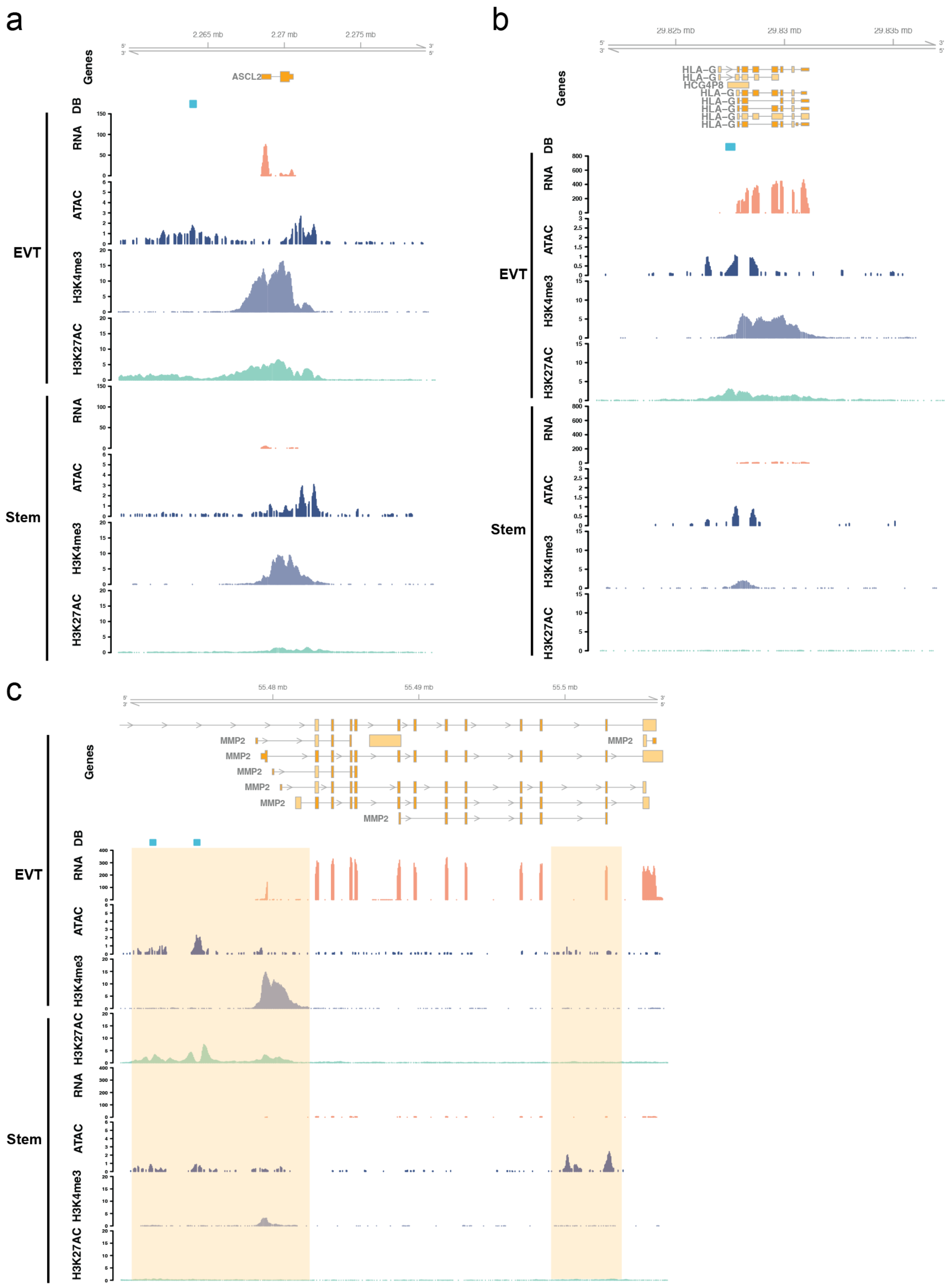

Supplementary Figure 6. Regulatory landscape near EVT cell-specific gene regions in CT27 cells. a-c) RNA-Seq, ATAC-Seq, histone 3 lysine 4 trimethylation (**H3K4me3**) and histone 3 lysine 27 acetylation (**H3K27ac**) ChIP-Seq assessments performed in EVT cells (top

panel), and stem state cells (bottom panel) are shown for regions surrounding *ASCL2* **(a)**, *HLA-G* **(b)**, and *MMP2* **(c)** in CT27 cells. Differentially bound (**DB**) regions are specific to the EVT cell state and are shown in blue rectangles. RNA-Seq (transcripts per million, y-axis), ATAC-Seq (counts per million mapped reads, y-axis), H3K4me3, and H3K27ac ChIP-Seq (counts per million mapped reads, y-axis) datasets are shown in individual tracks. Key regulatory regions are highlighted in yellow.

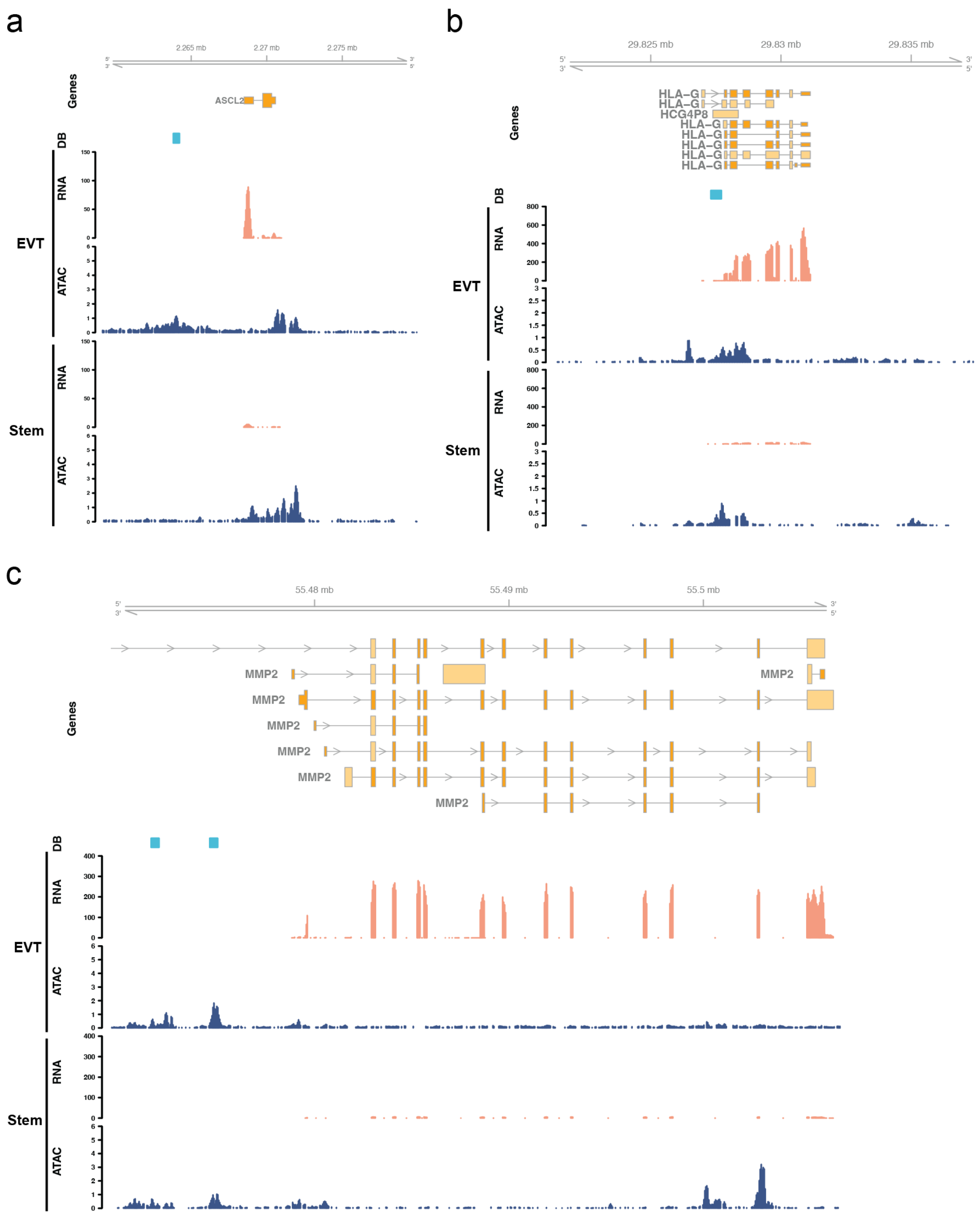

Supplementary Figure 7. Regulatory landscape near EVT-specific gene regions in CT29 cells. a-c) RNA-Seq and ATAC-Seq assessments performed in EVT cells (top panel), and stem state cells (bottom panel) are shown for regions surrounding *ASCL2* **(a)**, *HLA-G* **(b)**, and *MMP2* **(c)** in CT29 cells. Differentially bound (**DB**) regions are specific to the EVT cell state and are shown in blue rectangles. RNA-Seq (transcripts per million, y-axis) and ATAC-Seq (counts per million mapped reads, y-axis) datasets are shown in individual tracks.

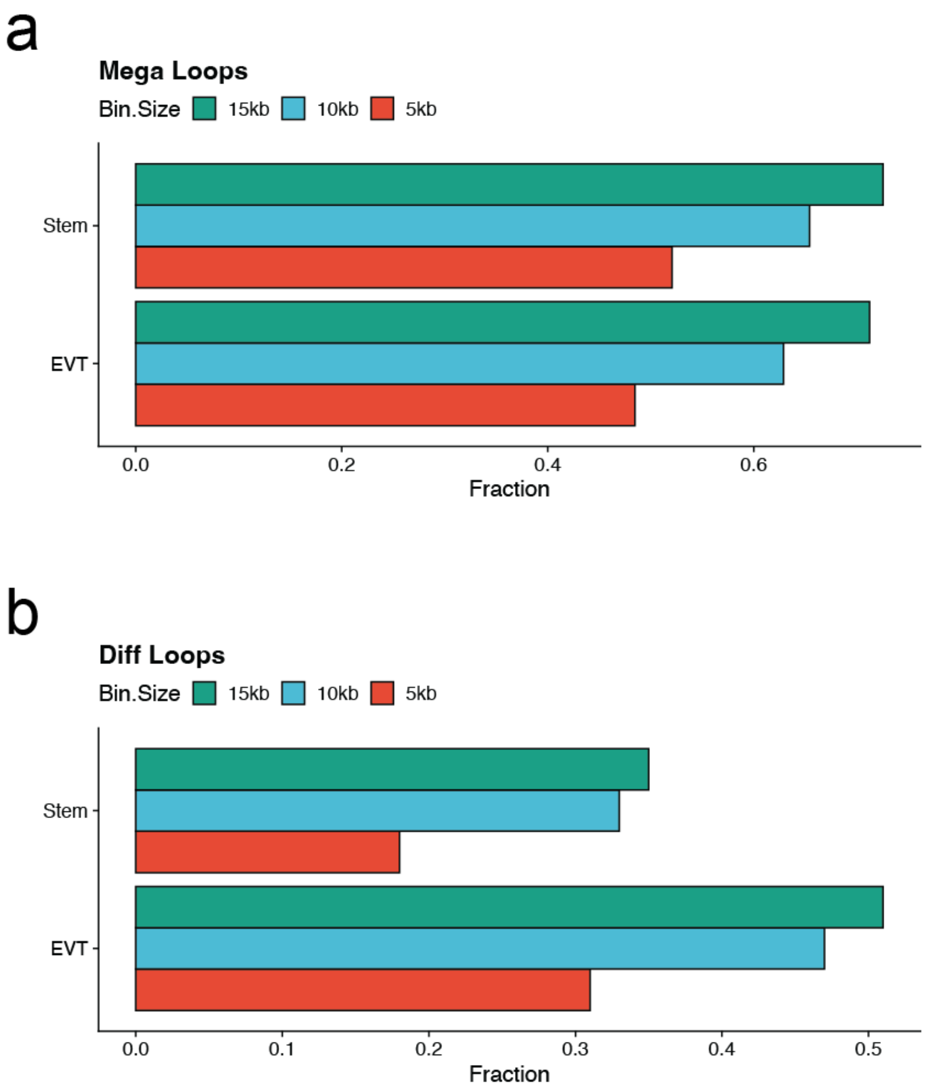

Supplementary Figure 8. Validation of long-range chromatin interactions in stem state and EVT differentiated cells. a-b) Bar graphs depicting the fraction (x-axis) of all Hi-C chromatin loops detected (**a**; Mega) or those unique (**b**; Diff) to either stem or EVT cell states in CT27 cell line that are also identified in the CT29 cell line within 5kb (red), 10kb (blue) or 15kb (green) of both anchors.

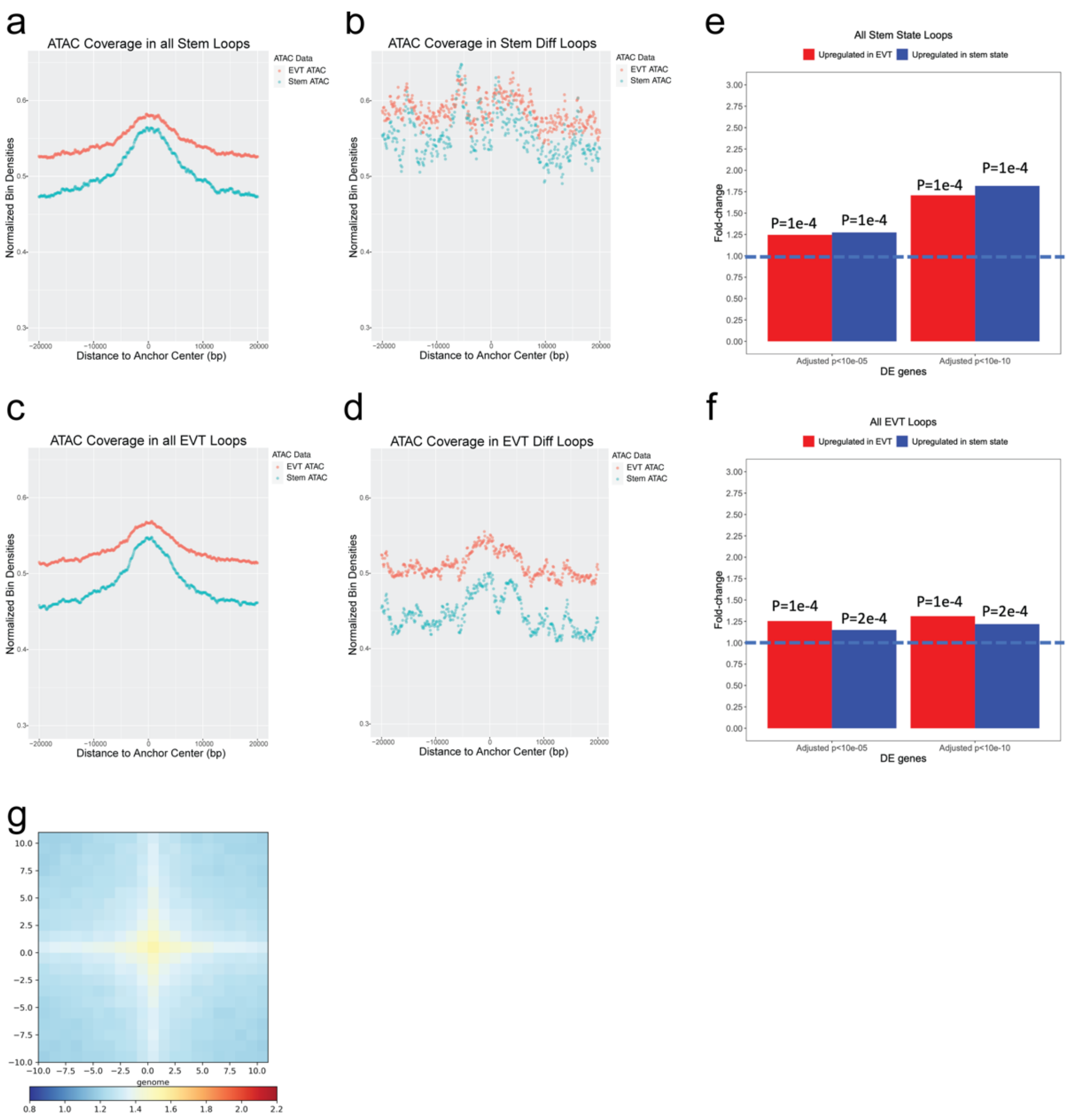

Supplementary Figure 9. Chromatin accessibility at loop anchors. a-d) Density plots showing distribution of open chromatin relative to the center of loop anchors (bp, x-axis). Normalized bin densities for every 10 million total reads (y-axis) as measured by ATAC-seq are shown for chromatin loops called in **a**) all Stem loops, **b**) Stem diff loops, **c**) all EVT loops and **d**) EVT diff loops**.** Proportion of all loop anchors identified in stem state **(e)** and EVT cells **(f)** that mapped nearby a gene (within 10kb) was calculated and enrichment of genes being significantly

upregulated as assessed by RNA-Seq in Stem state (blue bars) and EVT (red bars) cells, respectively, are presented as fold-change (y-axis). Dotted line corresponds to as many loop anchors being linked to significant vs non-significant genes, respectively. P-values are obtained from two-sided Fisher's exact test. g) Aggregate plot depicting EVT chromatin loop interactions at 9,334 EVT cell differentially accessed chromatin regions centered between total number of bins used in the submatrix (N=20). Enrichment is calculated based on transformation of observed to expected interactions and shown from low (0.8) to high (2.2) enrichment as illustrated using blue to red color scale.

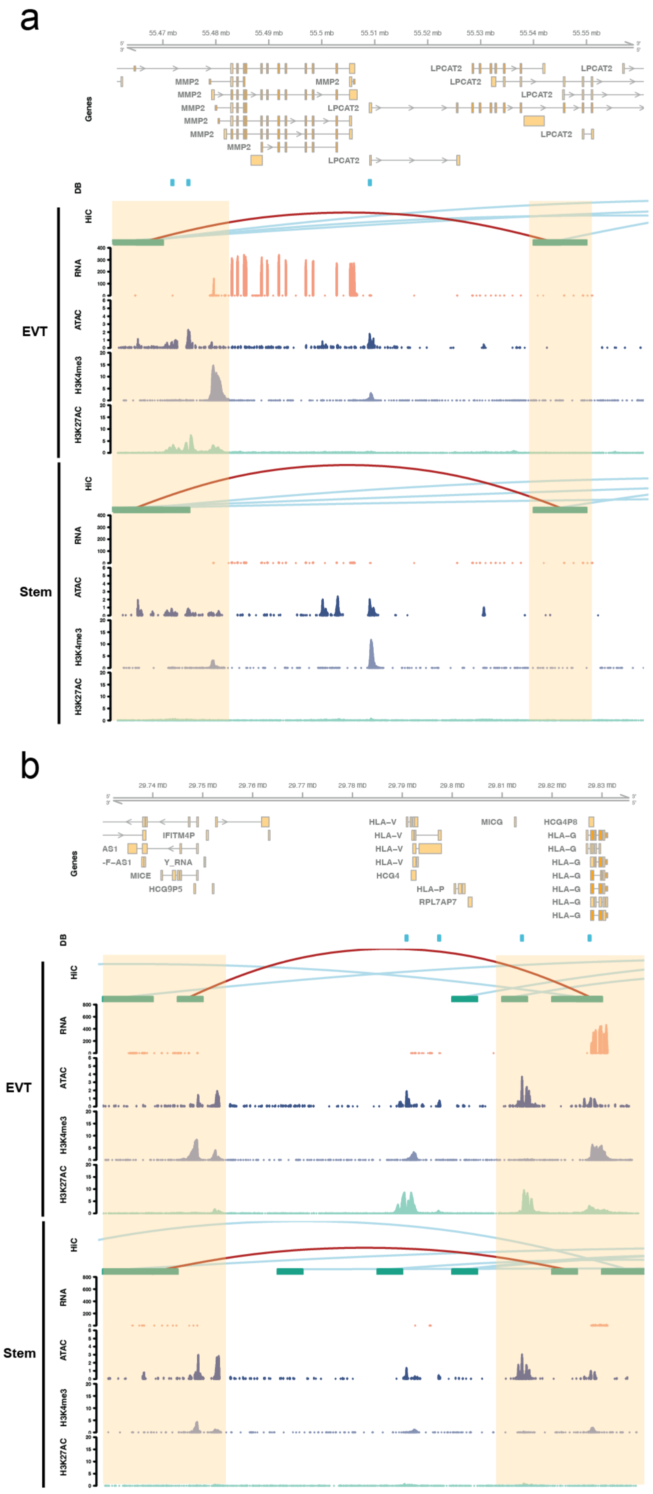

Supplementary Figure 10. Long-range chromatin interactions near *MMP2* and *HLA-G* in CT27 cells. a, b) Hi-C chromatin capture, RNA-Seq, ATAC-Seq, and H3K4me3 and H3K27ac ChIP-Seq assessments performed in EVT cells (top panel), and stem state cells (bottom panel)

are shown for regions surrounding *MMP2* **(a)** and *HLA-G* **(b)** in CT27 cells. Differentially bound (**DB**) regions are specific to the EVT cell state and are shown in blue rectangles. Hi-C loops (red, both loop anchors in view; blue, loop anchors out of view), RNA-Seq (transcripts per million, y-axis), ATAC-Seq (counts per million mapped reads, y-axis), H3K4me3, and H3K27ac ChIP-Seq (counts per million mapped reads, y-axis) datasets are shown in individual tracks. Key regulatory regions are highlighted in yellow.

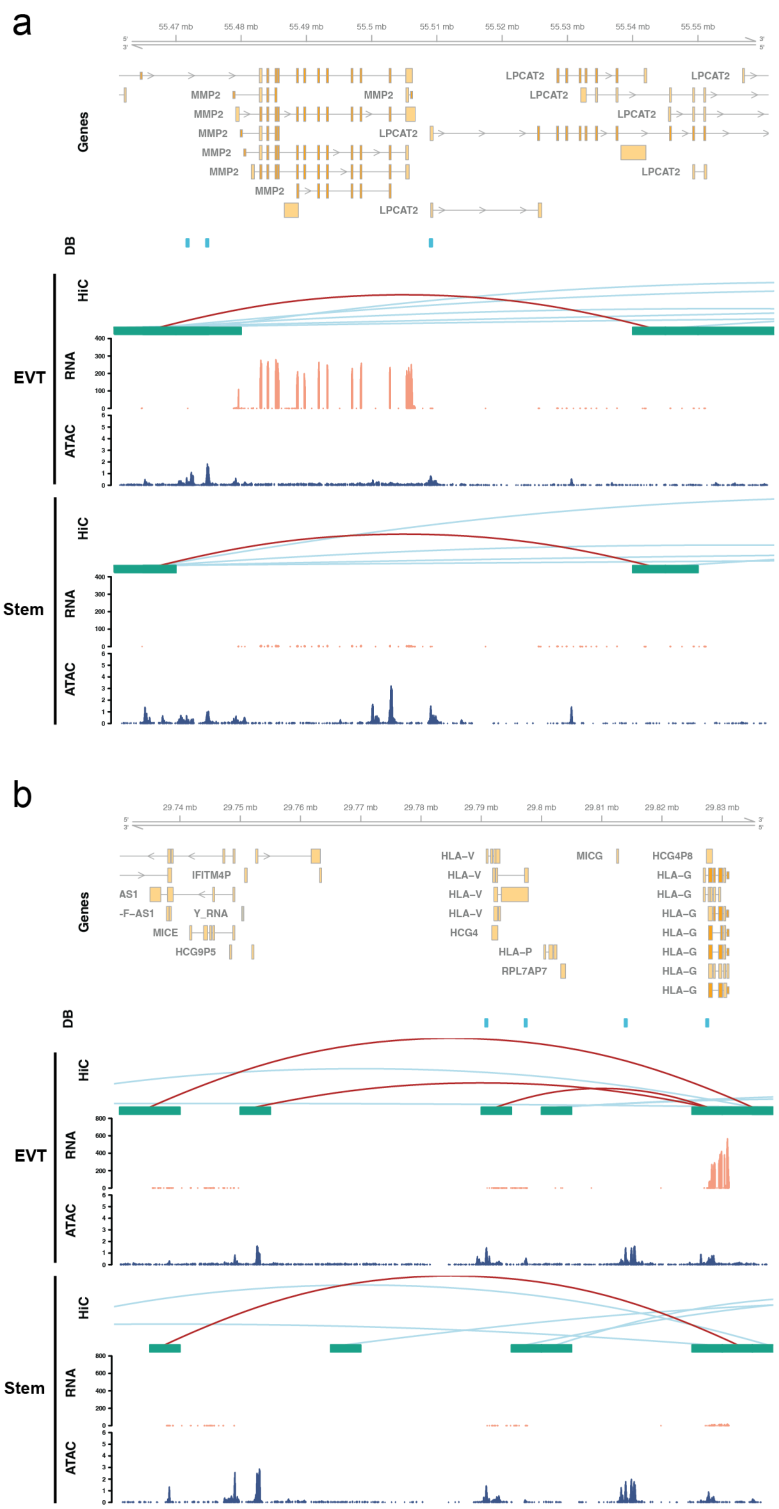

Supplementary Figure 11. Long-range chromatin interactions near *MMP2* and *HLA-G* in CT29 cells. a, b) Hi-C chromatin capture, RNA-Seq, and ATAC-Seq assessments performed in EVT cells (top panel), and stem state cells (bottom panel) are shown for regions surrounding

MMP2 **(a)** and *HLA-G* **(b)** in CT29 cells. Differentially bound (**DB**) regions are specific to the EVT cell state and are shown in blue rectangles. Hi-C loops (red, both loop anchors in view; blue, loop anchors out of view), RNA-Seq (transcripts per million, y-axis), and ATAC-Seq (counts per million mapped reads, y-axis) datasets are shown in individual tracks.

a

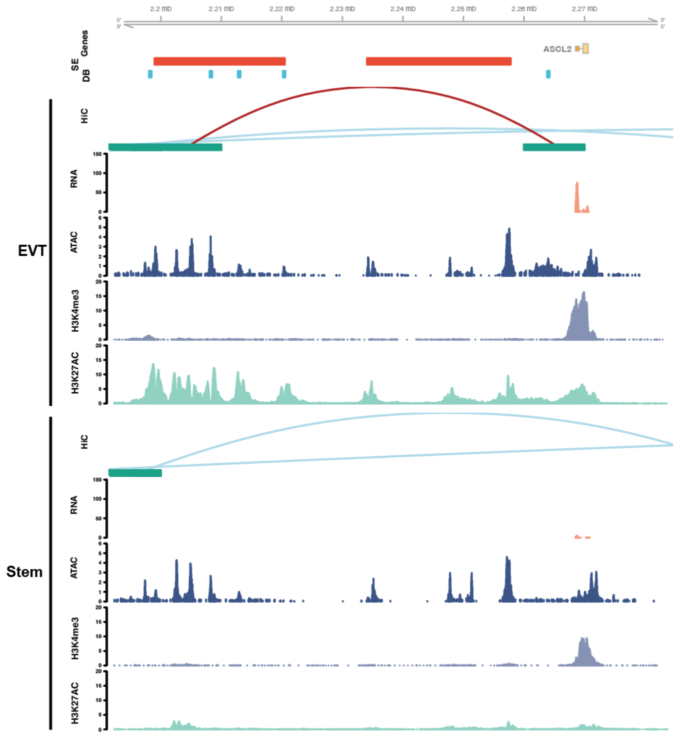

b

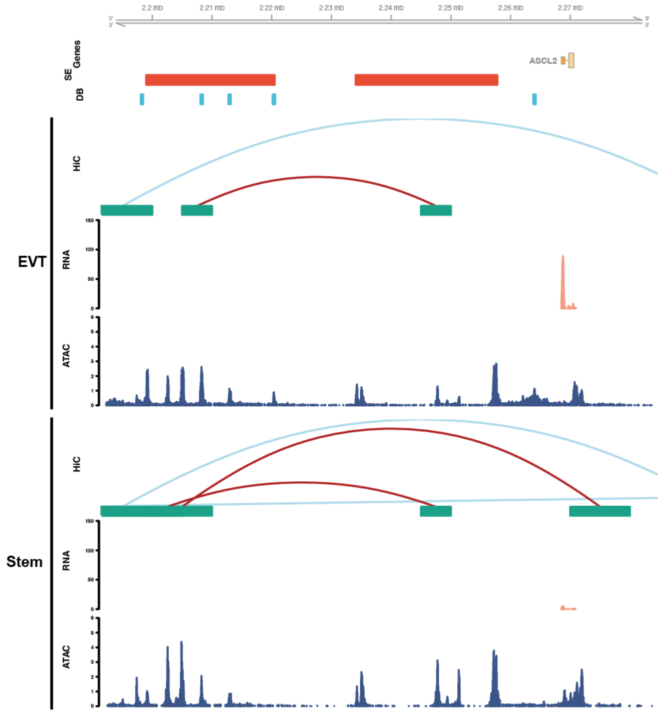

Supplementary Figure 12. Long-range chromatin interactions defining a unique regulatory landscape near *ASCL2* in EVT cells. a) Hi-C, RNA-Seq, ATAC-Seq, and

H3K4me3 and H3K27ac ChIP-Seq assessments performed in CT27 cells differentiated into EVT cells (top panel) or maintained in the stem state (bottom panel). Regulatory elements near *ASCL2* (yellow) are shown and include Hi-C loops (red, both loop anchors in view; blue, loop anchors out of view), RNA-Seq (transcripts per million, y-axis), ATAC-Seq (counts per million mapped reads, y-axis), H3K4me3, and H3K27ac ChIP-Seq (counts per million mapped reads, y-axis). All datasets are shown in individual tracks. Super-enhancers (**SE**, red) and differentially bound regions (**DB**, blue) are specific to the EVT cell state. **b)** Hi-C, RNA-Seq, and ATAC-Seq assessments performed in CT29 cells differentiated into EVT cells (top panel) or maintained in the stem state (bottom panel). Regulatory elements near *ASCL2* (yellow) are shown and include Hi-C loops (red, both loop anchors in view; blue, loop anchors out of view), RNA-Seq (transcripts per million, y-axis), and ATAC-Seq (counts per million mapped reads, y-axis). All datasets are shown in individual tracks. Super-enhancers (**SE**, red) and differentially bound regions (**DB**, blue) are specific to the EVT cell state.

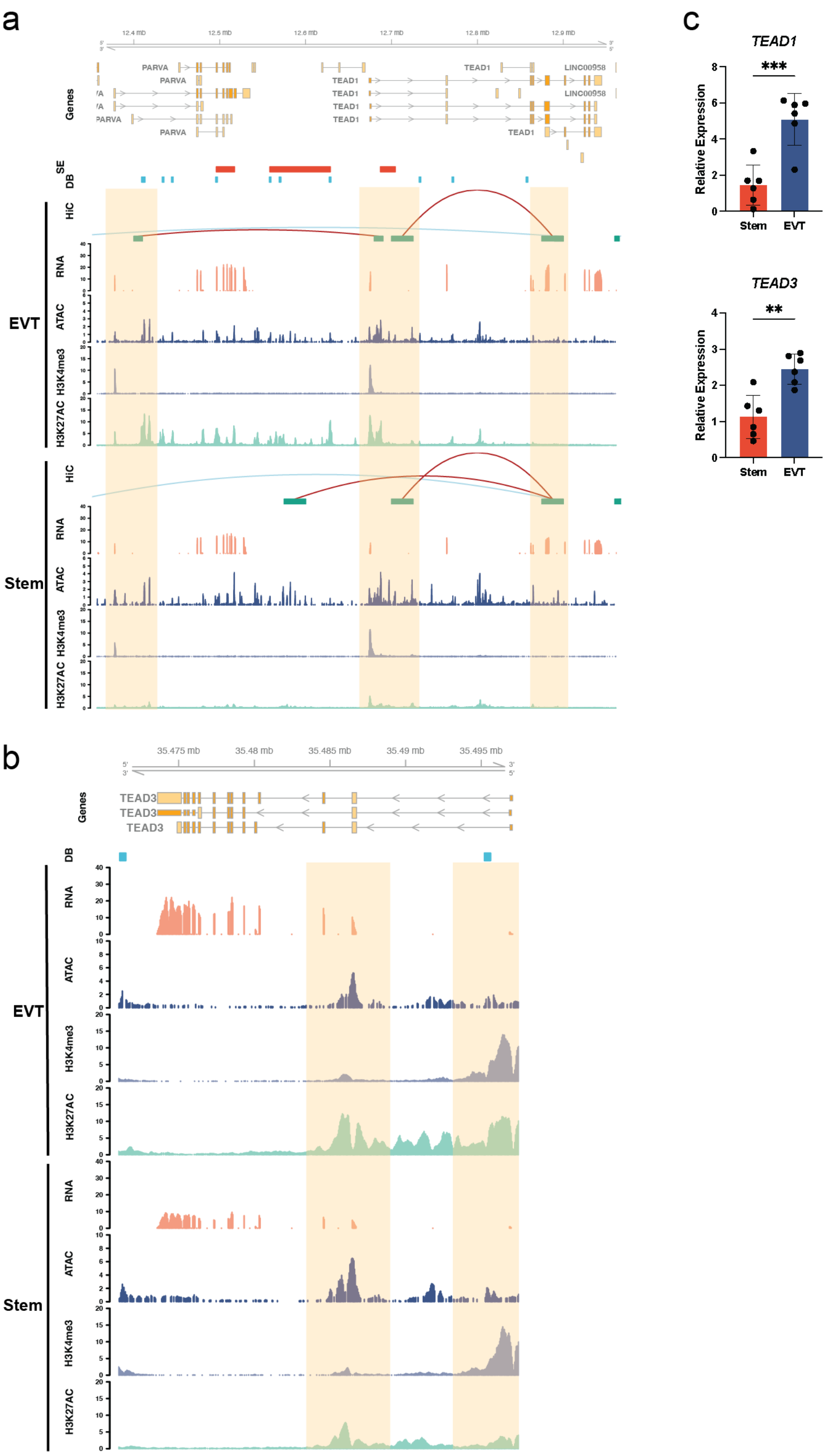

Supplementary Figure 13. Long-range chromatin interactions near genes of the TEAD transcription factor family in CT27 cells. Hi-C, RNA-Seq, ATAC-Seq, and H3K4me3 and

H3K27ac ChIP-Seq assessments performed in CT27 cells differentiated into EVT cells (top panel) or maintained in the stem state (bottom panel). Regulatory elements near *TEAD1* (**a**) and *TEAD3* (**b**) are shown and include Hi-C loops (red, both loop anchors in view; blue, loop anchors out of view), RNA-Seq (transcripts per million, y-axis), ATAC-Seq (counts per million mapped reads, y-axis), H3K4me3, and H3K27ac ChIP-Seq (counts per million mapped reads, y-axis). All datasets are shown in individual tracks. Super-enhancers (**SE**, red) and differentially bound regions (**DB**, blue) are specific to the EVT cell state. Key regulatory regions are highlighted in yellow. **c)** The relative expression of *TEAD1* (***p=0.0006), and *TEAD3* (**p=0.0012) in stem state (red) and EVT cells (blue) measured by RT-qPCR. Data were analyzed by unpaired t-test and are presented as mean values ± standard deviation (SD; n=6 individual biological replicates per group). Source data are provided as a Source Data file.

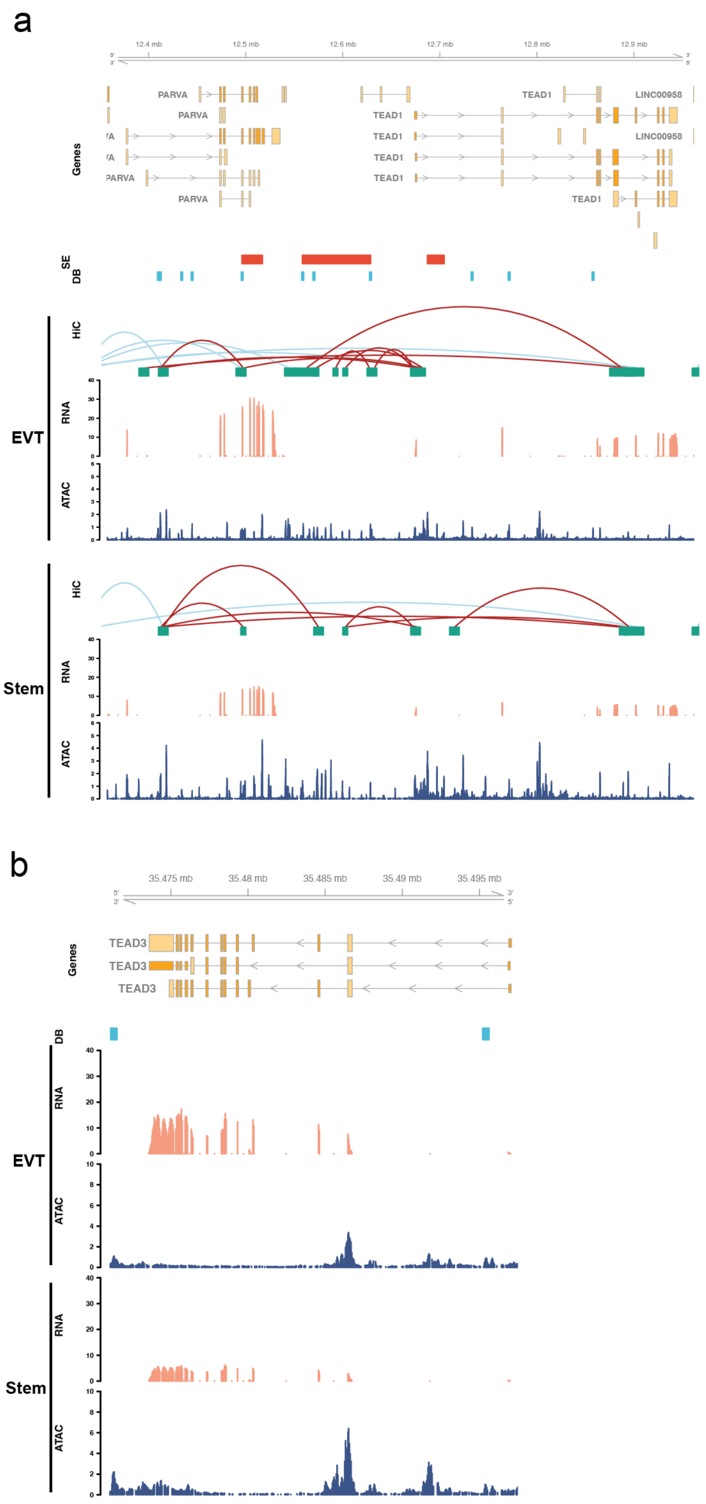

Supplementary Figure 14. Long-range chromatin interactions near genes of the TEAD transcription factor family in CT29 cells. Hi-C, RNA-Seq, and ATAC-Seq assessments

performed in CT29 cells differentiated into EVT cells (top panel) or maintained in the stem state (bottom panel). Regulatory elements near *TEAD1* (**a**) and *TEAD3* (**b**) are shown and include Hi-C loops (red, both loop anchors in view; blue, loop anchors out of view), RNA-Seq (transcripts per million, y-axis), and ATAC-Seq (counts per million mapped reads, y-axis). All datasets are shown in individual tracks. Super-enhancers (**SE**, red) and differentially bound regions (**DB**, blue) are specific to the EVT cell state.

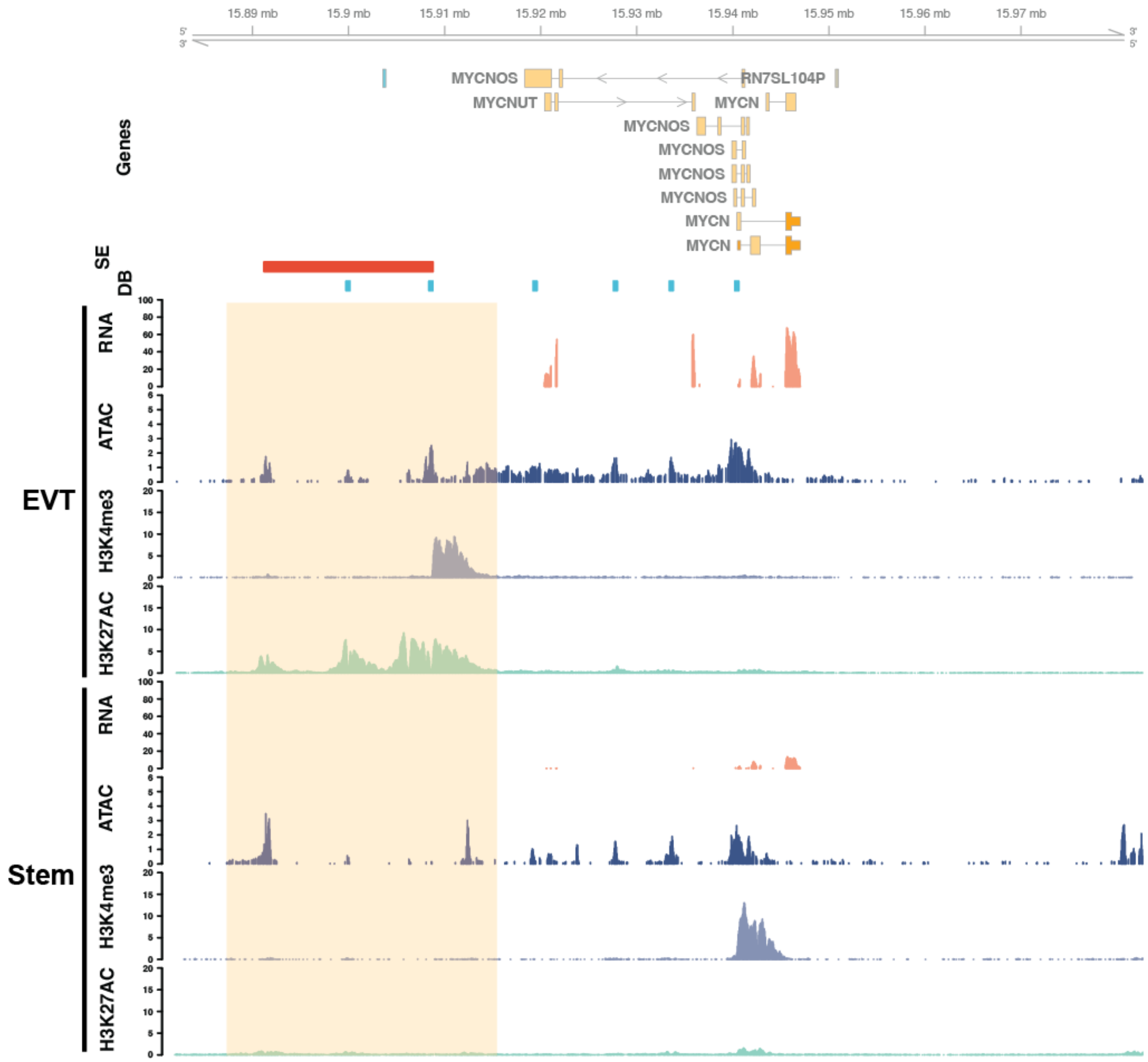

Supplementary Figure 15. Long-range chromatin interactions near *MYCN* in EVT cell differentiated CT27 cells. Hi-C, RNA-Seq, ATAC-Seq, and H3K4me3 and H3K27ac ChIP-Seq assessments performed in CT27 cells differentiated into EVT cells (top panel) or maintained in the stem state (bottom panel). Regulatory elements near *MYCN* are shown and include Hi-C loops (red, both loop anchors in view; blue, loop anchors out of view), RNA-Seq (transcripts per million, y-axis), ATAC-Seq (counts per million mapped reads, y-axis), H3K4me3, and H3K27ac ChIP-Seq (counts per million mapped reads, y-axis). All datasets are shown in individual tracks. Super-enhancers (**SE**, red) and differentially bound regions (**DB**, blue) are specific to the EVT cell state. Key regulatory regions are highlighted in yellow.

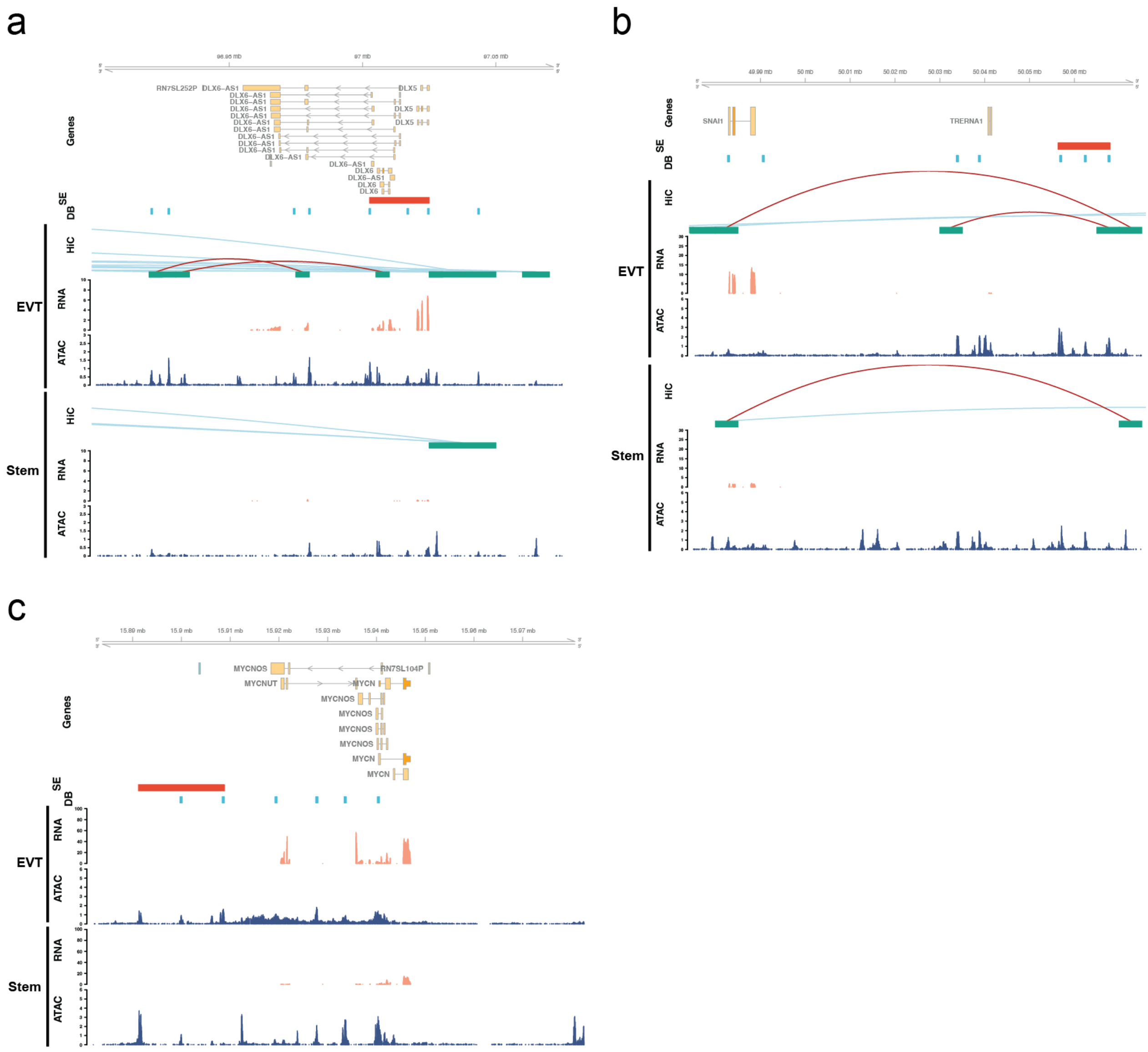

Supplementary Figure 16. Long-range chromatin interactions for transcriptional regulators of EVT cell differentiation in CT29 cells. Hi-C, RNA-Seq, and ATAC-Seq assessments performed in CT29 cells differentiated into EVT cells (top panel) or maintained in the stem state (bottom panel). Regulatory elements near *DLX5/DLX6* (**a**), *SNAI1* (**b**), and *MYCN* (**c**) are shown and include Hi-C loops (red, both loop anchors in view; blue, loop anchors out of view), RNA-Seq (transcripts per million, y-axis), and ATAC-Seq (counts per million mapped reads, y-axis). All datasets are shown in individual tracks. Super-enhancers (**SE**, red) and differentially bound regions (**DB**, blue) are specific to the EVT cell state.

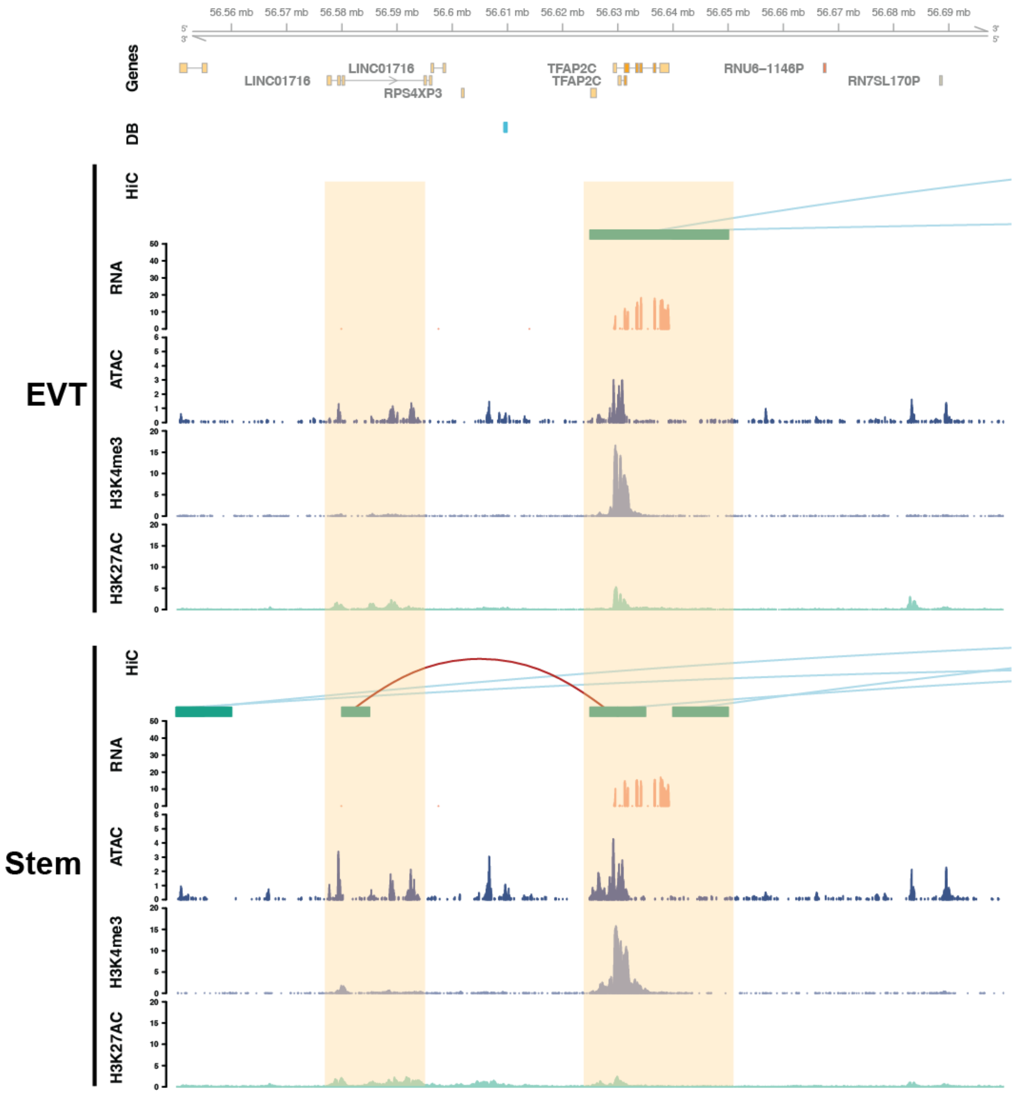

Supplementary Figure 17. Regulatory landscape of *TFAP2C* in CT27 cells. Hi-C, RNA-Seq, ATAC-Seq, and H3K4me3 and H3K27ac ChIP-Seq assessments performed in CT27 cells differentiated into EVT cells (top panel) or maintained in the stem state (bottom panel). Regulatory elements near *TFAP2C* are shown and include Hi-C loops (red, both loop anchors in view; blue, loop anchors out of view), RNA-Seq (transcripts per million, y-axis), ATAC-Seq (counts per million mapped reads, y-axis), K4me3, and K27ac ChIP-Seq (counts per million mapped reads, y-axis). All datasets are shown in individual tracks. Differentially bound regions (**DB**, blue) are specific to the EVT cell state. Key regulatory regions are highlighted in yellow.

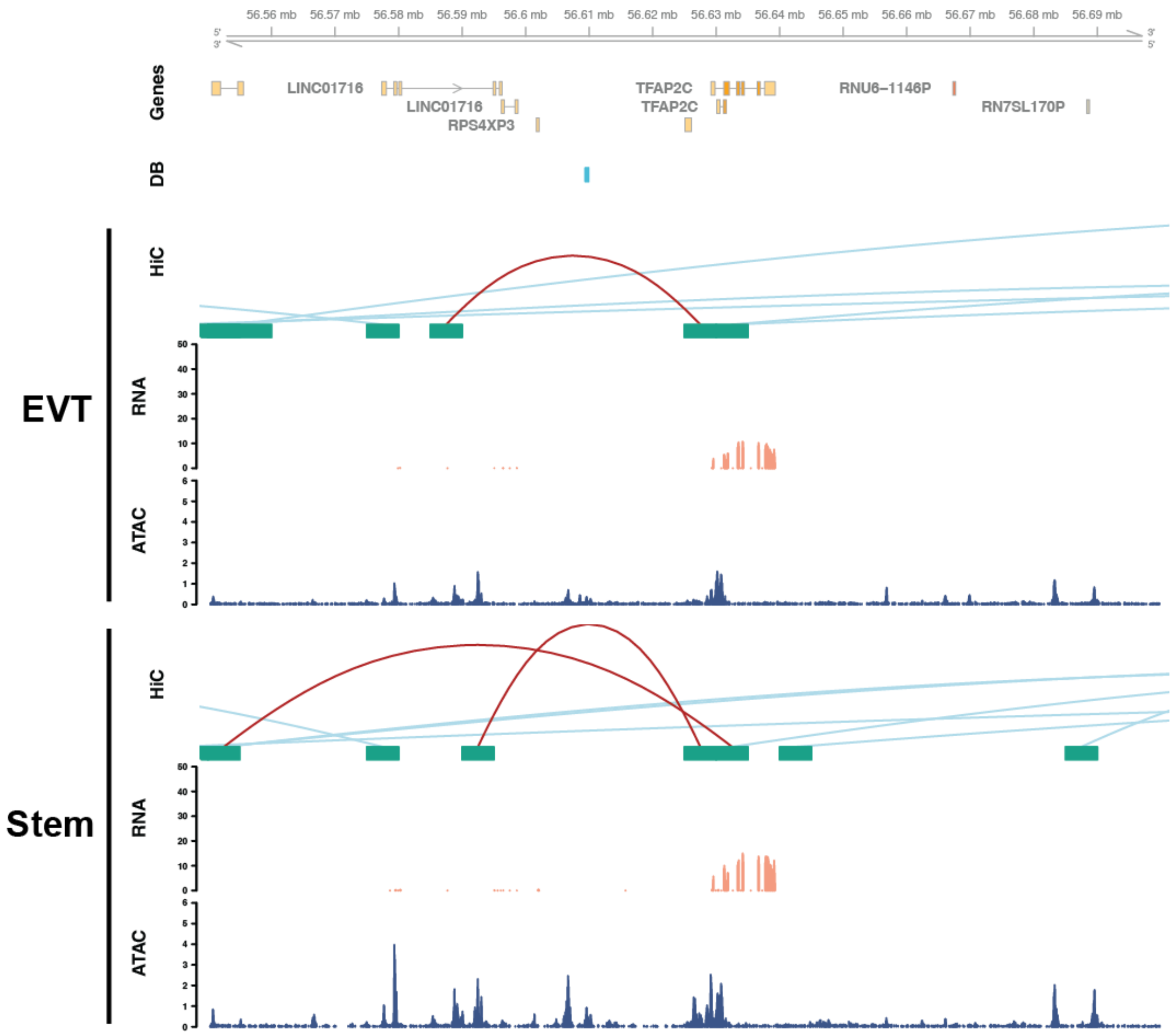

Supplementary Figure 18. Long-range chromatin interactions near *TFAP2C* in CT29 cells. Hi-C, RNA-Seq, and ATAC-Seq assessments performed in CT29 cells differentiated into EVT cells (top panel) or maintained in the stem state (bottom panel). Regulatory elements near *TFAP2C* are shown and include Hi-C loops (red, both loop anchors in view; blue, loop anchors out of view), RNA-Seq (transcripts per million, y-axis), and ATAC-Seq (counts per million mapped reads, y-axis). All datasets are shown in individual tracks. Differentially bound regions (**DB**, blue) are specific to the EVT cell state.

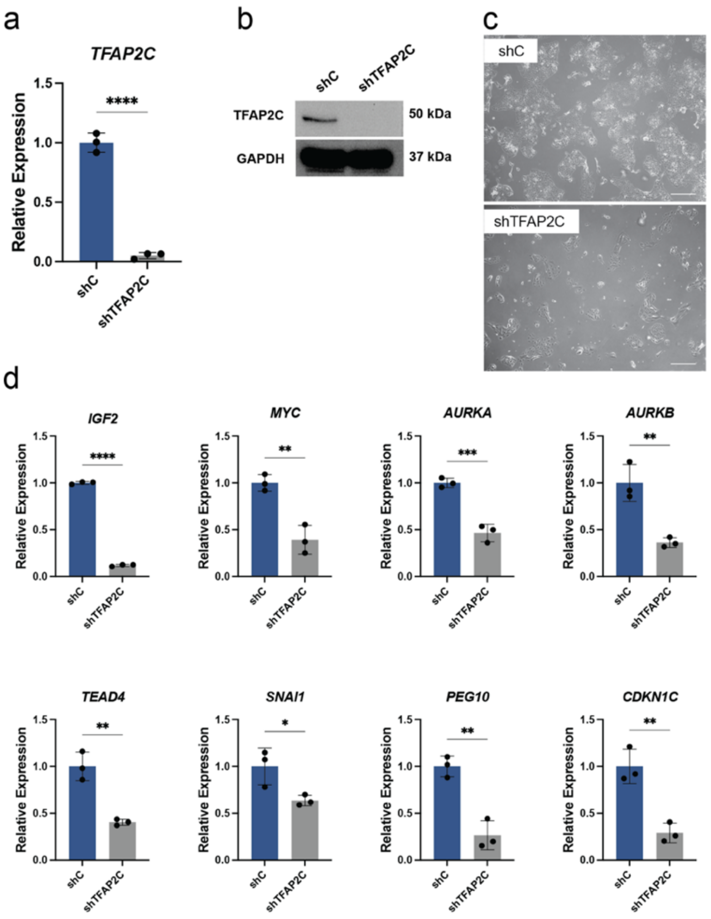

Supplementary Figure 19. Examination of the impact of trophoblast cell differentiation in CT29 cells upon *TFAP2C* disruption. a) Relative expression of *TFAP2C* normalized to *POLR2A* in stem state cells transduced with lentivirus containing a control shRNA (**shC**) or a *TFAP2C*-specific shRNA (**shTFAP2C**; ****p<0.0001). **b)** TFAP2C (50 kilodaltons (kDa)) and GAPDH (37 kDa) proteins in stem state cells transduced with shC or shTFAP2C and assessed by western blot. **c)** Phase contrast images of stem state cells transduced with shC or shTFAP2C. Scale bar represents 500 µm. **d)** RT-qPCR measurement of *IGF2* (p<0.0001), *MYC* (p=0.004), *AURKA* (p=0.0009), *AURKB* (p=0.0058), *TEAD4* (p=0.0027), *SNAI1* (p=0.036), *PEG10* (p=0.0026), and *CDKN1C* (p=0.0043) in stem state cells transduced with shC or shTFAP2C. Data in panels a and d were analyzed by unpaired t-test and are presented as mean values ± standard deviation (SD; (n=3 biologically independent replicates per group). Source data are provided as a Source Data file.

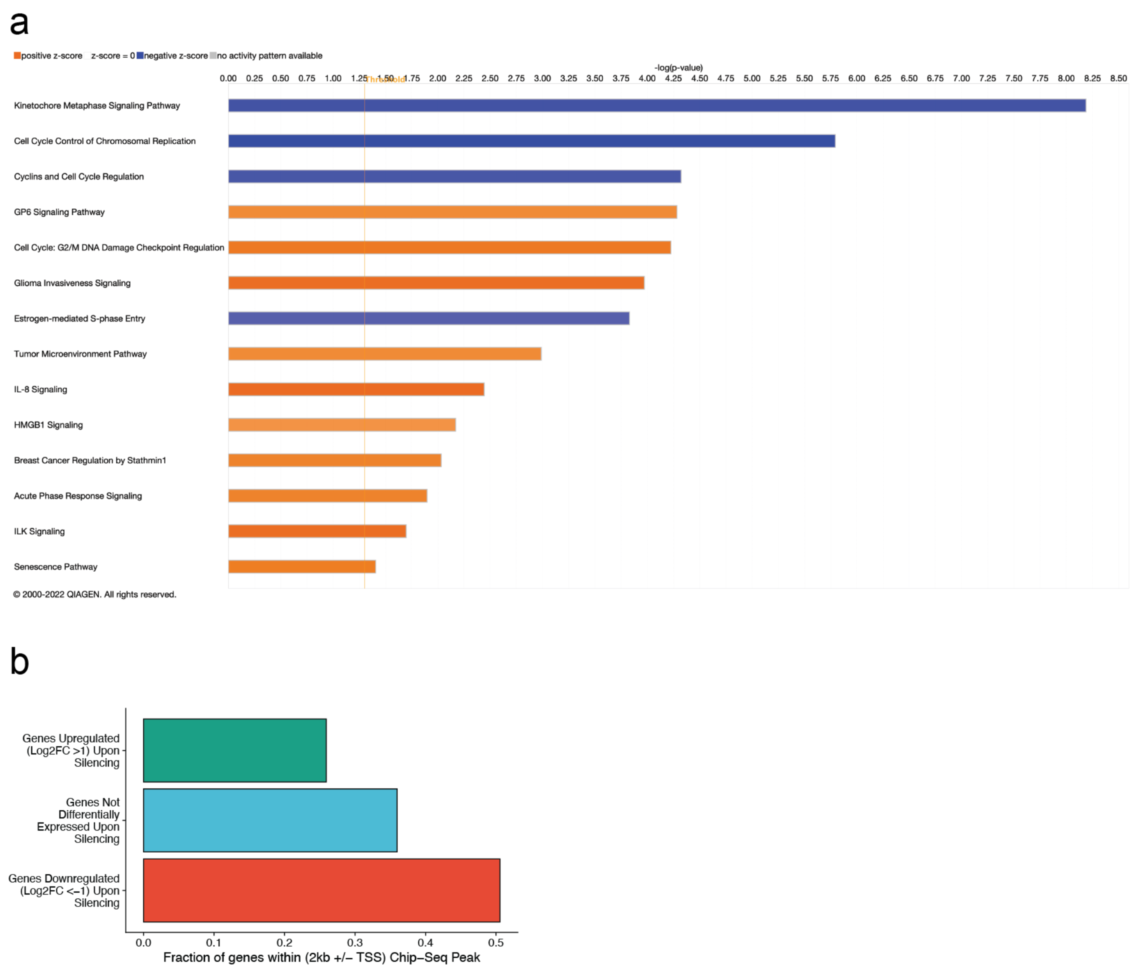

Supplementary Figure 20. ***TFAP2C*** **regulation of genes linked to cell cycle control. a)** Ingenuity pathway analysis of the top 500 genes regulated by *TFAP2C* (upregulated or downregulated). The top functional pathways (z-score >2) included kinetochore metaphase signaling, cell cycle control of chromosomal replication, as well as cyclins and cell cycle regulation. Positive (orange) and negative (blue) z-scores are presented as histogram bars and -log(B-H p-value) are represented on the x-axis. **b)** Bar graph depicting the fraction of upregulated genes upon *TFAP2C* silencing ($Log_2FC>1$, green bar), not differentially expressed upon *TFAP2C* silencing (blue bar) and downregulated genes upon *TFAP2C* silencing ($Log_2FC<1$, red bar) overlapping a TFAP2C ChIP-Seq peak mapping within 2 kb of the transcription start site (**TSS**) of each gene.

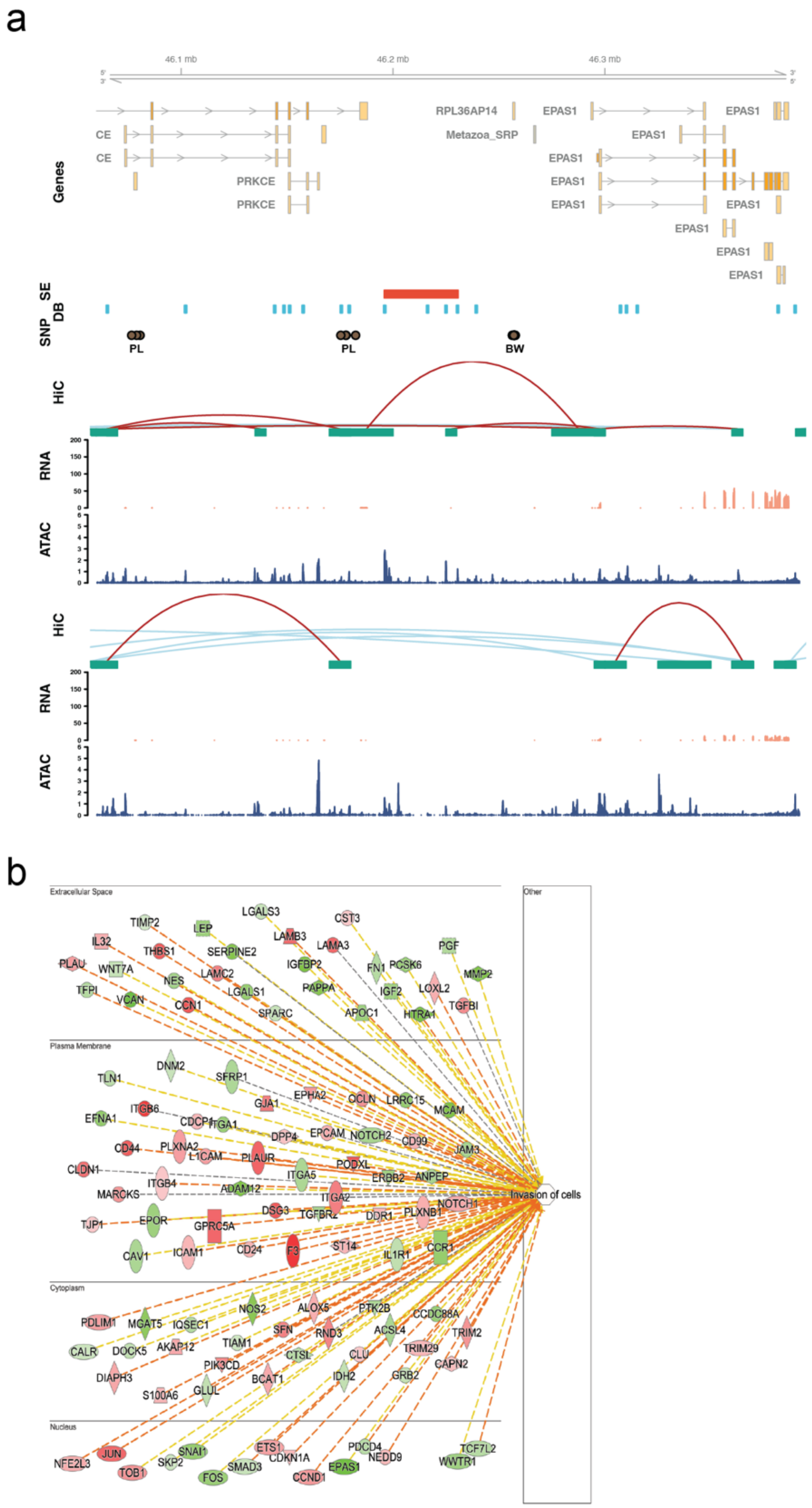

Supplementary Figure 21. Functional investigation of *EPAS1* on EVT cell differentiation.

a) Hi-C, RNA-Seq, and ATAC-Seq assessments performed in CT29 cells differentiated into EVT

cells (top panel) or maintained in the stem state (bottom panel). Regulatory elements near *EPAS1* are shown and include Hi-C loops (red, both loop anchors in view; blue, loop anchors out of view), RNA-Seq (transcripts per million, **TPM**, y-axis), and ATAC-Seq (counts per million mapped reads, **CPM**, y-axis). All datasets are shown in individual tracks. Super-enhancers (SE, red) and differentially bound regions (**DB**, blue) are specific to the EVT cell state. SNPs associated with pregnancy loss (PL) or birth weight (BW) in large genome-wide association studies (GWAS) are shown in brown circles. **b)** Network schematic depicting results from Ingenuity Pathway Analysis performed on top 500 differentially expressed genes (upregulated or downregulated) by *EPAS1,* in CT27 cells of which 209 genes was annotated to the biological function “Migration of Cells” (two-sided Fisher’s exact test, p=8.3E-49). The most significant biological function associated with this subset was “Invasion of Cells” (two-sided Fisher’s exact test, p=5.3E-70). The biological function is displayed as a network with subcellular layout with gene targets as nodes and the node shapes indicate the gene’s primary function. Node colors indicate increased (red) or decreased (green) expression of the genes following *EPAS1* disruption.

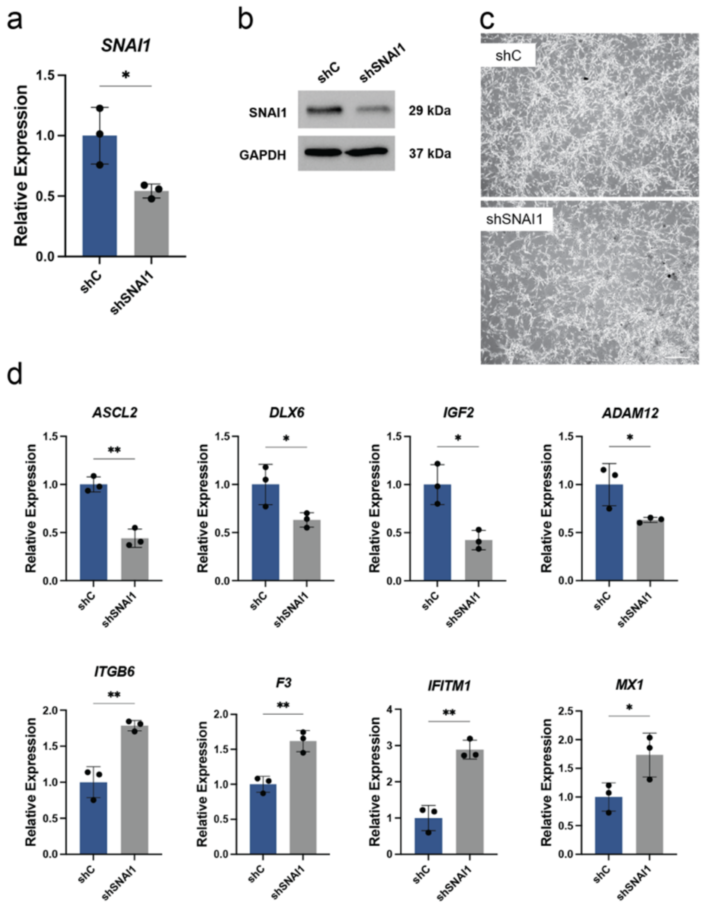

Supplementary Figure 22. *SNAI1* disruption impairs EVT cell differentiation in CT29 cells. a) Relative expression of *SNAI1* normalized to *POLR2A* in EVT cells differentiated from stem state cells transduced with lentivirus containing a control shRNA (**shC**) or a *SNAI1*-specific shRNA (**shSNAI1**; *p=0.00306). **b)** SNAI1 (29 kDa) and GAPDH (37 kDa) proteins in EVT cells differentiated from stem state cells transduced with shC or shSNAI1 and assessed by western blot. **c)** Phase contrast images of EVT cells differentiated from stem state cells transduced with shC or shSNAI1. Scale bar represents 500 µm. **d)** RT-qPCR measurement of *ASCL2* (p=0.0014), *DLX6* (p=0.0457), *IGF2* (p=0.0125), *ADAM12* (p=0.0449), *ITGB6* (p=0.0039), *F3* (p=0.0048), *IFITM1* (p=0.0017), and *MX1* (p=0.0491) in EVT cells differentiated from stem state cells transduced with shC or shSNAI1. Data in panels a and d were analyzed by unpaired t-test and are presented as mean values ± standard deviation (SD; (n=3 biologically independent replicates per group). Source data are provided as a Source Data file.

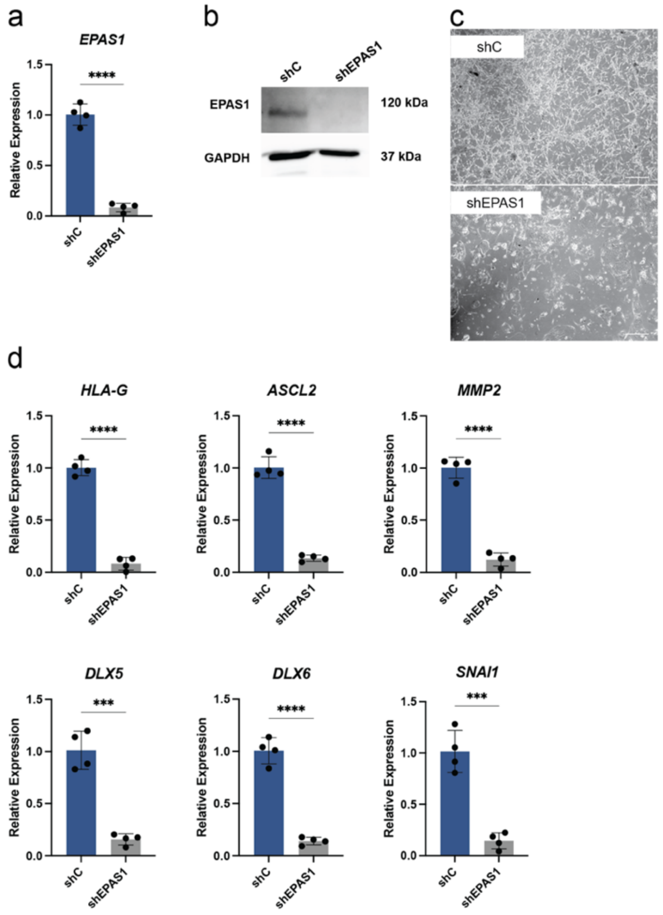

Supplementary Figure 23. *EPAS1* disruption impairs EVT cell differentiation in CT29 cells. a) Relative expression of *EPAS1* normalized to *POLR2A* in EVT cells differentiated from stem state cells transduced with lentivirus containing a control shRNA (**shC**) or a *EPAS1*-specific shRNA (**shEPAS1**; ****p<0.0001). **b)** HIF2a (29 kDa) and GAPDH (37 kDa) proteins in EVT cells differentiated from stem state cells transduced with shC or shEPAS1 and assessed by western blot. **c)** Phase contrast images of EVT cells differentiated from stem state cells transduced with shC or shEPAS1. Scale bar represents 500 µm. **d)** RT-qPCR measurement of *HLA-G* (p<0.0001), *ASCL2* (p<0.0001), *MMP2* (p<0.0001), *DLX5* (p=0.0001), *DLX6* (p<0.0001), and *SNAI1* (p=0.0002), in EVT cells differentiated from stem state cells transduced with shC or shEPAS1. Data in panels a and d were analyzed by unpaired t-test and are presented as mean values ± standard deviation (SD; (n=4 biologically independent replicates per group). Source data are provided as a Source Data file.

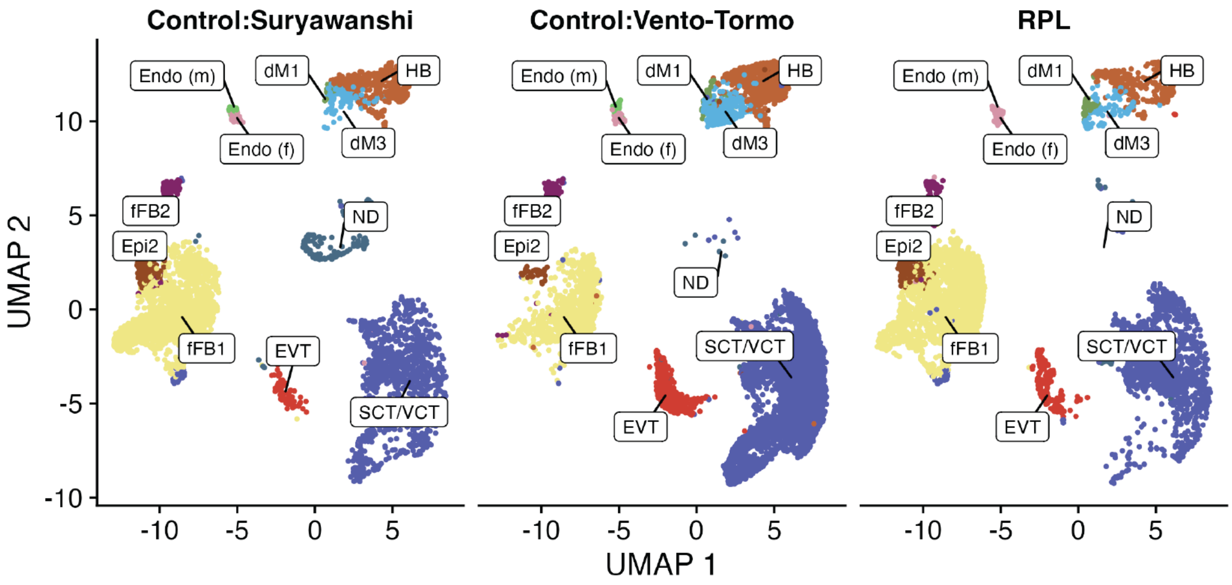

Supplementary Figure 24. Human placenta gene expression profiles from combined single cell datasets of control and RPL. UMAP plots depicting cell clustering from two publicly available[2,3] (control; left and middle) and one newly generated (**RPL**; right) single-cell RNA sequencing datasets. Cell types are inferred using marker genes published previously[3] as decidual macrophages (**dM**), fetal (**Endo f**) and maternal endothelial cells (**Endo m**), epithelial glandular cells (**Epi**), extravillous trophoblast cells (**EVT**), fetal fibroblasts (**FB**), Hofbauer cells (**HB**), syncytiotrophoblast and villous cytotrophoblast (**SCT/VCT**) and cells not determined (**ND**).

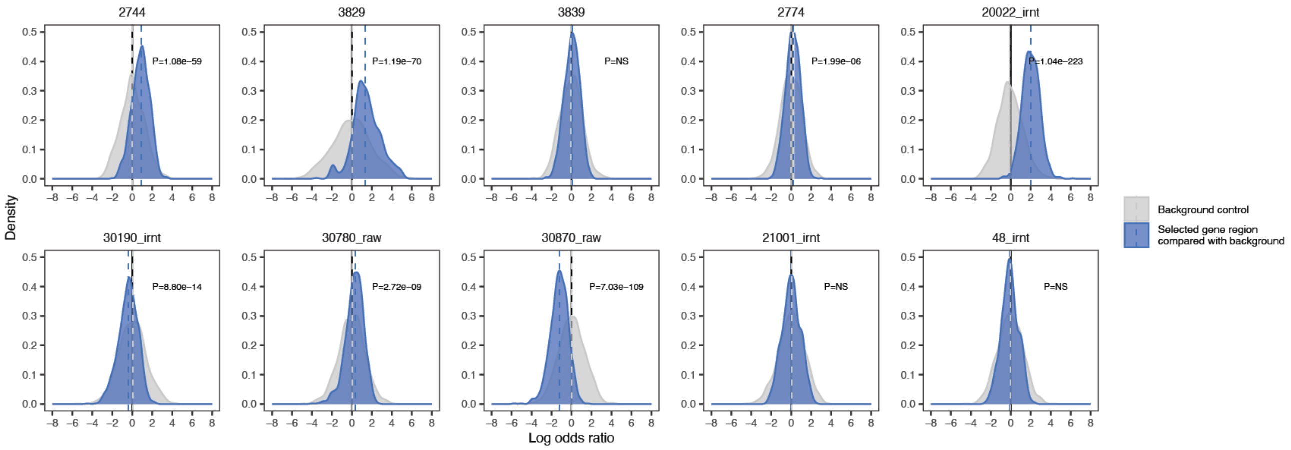

Supplementary Figure 25. Enrichment analysis of genetic variants near *EPAS1*associated with pregnancy phenotypes in the UK Biobank. Density plot of the distribution of log transformation of odds ratios (y-axis) for association of genetic variants near *EPAS1* (chr2:46,150,000-46,450,000) and pregnancy phenotypes in UK Biobank compared with genetic variants in randomly selected matched window (blue) with the median odds ratio indicated by dashed blue line. Indicated p-value represent the significance of the odds ratio distribution compared to a null distribution (gray) estimated by the two-sided Wilcoxon test. Enrichment analysis was performed on five pregnancy-related phenotypes (2744=Birth weight of first child; 3829=Number of stillbirths; 3839=Number of spontaneous miscarriages; 2774= Ever had stillbirth, spontaneous miscarriage or termination; 20022_irnt=Birth weight) and five randomly selected phenotypes as control (30190_irnt=Monocyte percentage; 30780_raw= LDL direct; 30870_raw=Triglycerides; 21001_irnt= Body mass index (BMI); 48_irnt= Heel bone mineral density (BMD).

Supplementary Table 1: Phenotypes from UK Biobank GWAS used for Enrichment Analysis

PhenotypeID	Description	Variable_type	Source	N_non_missing	N_missing	N_controls	N_cases
2744	Birth weight of first child	ordinal	phesant	155,202	38,972		
3829	Number of stillbirths	ordinal	phesant	60,453	133,721		
3839	Number of spontaneous miscarriages	ordinal	phesant	60,300	133,874		
2774	Ever had stillbirth, spontaneous miscarriage, or termination	binary	phesant	191,252	2,922	130,687	60,565
20022_irnt	Birth weight	continuous_irnt	phesant	205,475	155,719		

Supplementary Table 2: Placental samples included in single-cell RNA analysis

Cohort	Sample ID	GA	Cause of Pregnancy Loss	Maternal age (years)	Maternal BMI (kg/m2)	Fetal Chromosome Analysis Results	Fetal Microarray Analysis Results	# of Captures	Cells loaded per capture
RPL	cmh002898-01	6w1d	Miscarriage	32	29.9	46, XY	arr(1-22)x2,(X,Y)x1	1	16500
RPL	cmh003068-01	8w0d	Miscarriage	36	19.2	46,XY	arr(1-22)x2,(X,Y)x1	2	16500
RPL	cmh003961-01	6w4d	Miscarriage	40	21.1	46,XY	arr(1-22)x2,(X,Y)x1	1	16500
Normal Control (Vento-Tormo)*	D10	8w	Elective termination	NA	NA	NA	NA	2	NA
Normal Control (Vento-Tormo)*	D12	8w	Elective termination	NA	NA	NA	NA	1	NA
Normal Control (Suryawanshi)**	Placenta_23	7w6d	Elective termination	NA	NA	46,XY	NA	1	NA

*D6, D7 excluded as no single-cell RNA was done on placental tissue, only decidua; D8 excluded due to fetal diagnosis of Edwards syndrome; D9 excluded due to gestational age (10w); D11 excluded due to medical termination of pregnancy. **Placenta_17 excluded due to poor quality; Placenta_5, Placenta_10, Placenta_11, Placenta_12, Placenta_20 excluded because scRNA-seq libraries were generated with Drop-seq protocol; Placenta_21, Placenta_22 excluded as no single-cell RNA was done on placental tissue.

Supplementary Table 3: Short hairpin RNA (shRNA) sequences

**shRNA Target**	**Oligo #**	**Species**	**Sequence**
TFAP2C	1	Human	CCGGTCCTATGTCTGTGAAGCCGAATCTCGAGATTCGGCTTCACAGACATAGGTTTTTG
TFAP2C	2	Human	AATTCAAAAACCTATGTCTGTGAAGCCGAATCTCGAGATTCGGCTTCACAGACATAGGA
SNAI1	1	Human	CCGGTGCAGGACTCTAATCCAGAGTTCTCGAGAACTCTGGATTAGAGTCCTGCTTTTTG
SNAI1	2	Human	AATTCAAAAAGCAGGACTCTAATCCAGAGTTCTCGAGAACTCTGGATTAGAGTCCTGCA
EPAS1	1	Human	CCGGTCAGTACCCAGACGGATTTCAACTCGAGTTGAAATCCGTCTGGGTACTGTTTTTG
EPAS1	2	Human	AATTCAAAAACAGTACCCAGACGGATTTCAACTCGAGTTGAAATCCGTCTGGGTACTGA

Supplementary Table 4: RT-qPCR primer sequences

Target	Species	Forward	Reverse
ASCL2	Human	GCACCAACACTTGGAGATTTT	AATGGATTCTCTGTGCCCTTAG
B2M	Human	TTCTGGCCTGGAGGCTATC	TCAGGAAATTTGACTTTCCATTC
EPAS1	Human	GTCAGCCCACAAGGTGTCA	CACAGTCATATCTGGTCAGTTCG
HLA-G	Human	CCACCACCCTGTCTTTGACTAT	ACGTCCTGGGTCTGGTCCT
ITGA1	Human	AATTGGCTCTAGTCACCATTGTT	CAAATGAAGCTGCTGACTGGT
MMP2	Human	TGGCACCCATTTACACCTACAC	ATGTCAGGAGAGGCCCCATAGA
POLR2A	Human	TCCGTATTCGCATCATGAAC	TCATCCATCTTGTCCACCAC
SNAI1	Human	TACAGCGAGCTGCAGGACT	ATCTCCGGAGGTGGGATG
TEAD1	Human	CTCCATTGGCACAACCAAGC	CATGCCCAATGTGCACGAAG
TEAD3	Human	GGGAAGACTCGGACGAGAAAA	TGATGCCAACCTGGTACTCC
TEAD4	Human	CAGGTGGTGGAGAAAGTTGAGA	GTGCTTGAGCTTGTGGATGAAG
TFAP2C	Human	AAGCCGCTCATGTGACTCTC	TCGGCTTCACAGACATAGGC

References

1. Okae, H. *et al.* Derivation of human trophoblast stem cells. *Cell Stem Cell* **22**, 50-63.e6 (2018).
2. Suryawanshi, H. *et al.* A single-cell survey of the human first-trimester placenta and decidua. *Sci. Adv.* **4**, eaau4788 (2018).
3. Vento-Tormo, R. *et al.* Single-cell reconstruction of the early maternal-fetal interface in humans. *Nature* **563**, 347–353 (2018).
4. Warrington, N. M. *et al.* Maternal and fetal genetic effects on birth weight and their relevance to cardio-metabolic risk factors. *Nat. Genet.* **51**, 804–814 (2019).
