# Supplementary material for: Extravillous trophoblast cell lineage development is associated with active remodeling of the chromatin landscape
Source: Nat Commun. 2023 Aug 10;14:4826. doi: 10.1038/s41467-023-40424-5 (PMC10415281; doi:10.1038/s41467-023-40424-5)
Supplement: Supplementary file 3 — Description of Additional Supplementary Files [file 41467_2023_40424_MOESM3_ESM.pdf]

Description of Additional Supplementary Files

File Name: Supplementary Data 1

Description: Sample information

File Name: Supplementary Data 2

Description: Differential gene expression in EVT cells vs Stem state cells. P values estimated by a Wald test followed by Benjamini and Hochberg procedure to adjust for multiple testing implemented in DESeq2.

File Name: Supplementary Data 3a

Description: Chromatin accessibility in stem state cells. P values for called peaks estimated by a Poisson model followed by Benjamini and Hochberg procedure to adjust for multiple testing implemented in MACS3.

File Name: Supplementary Data 3b

Description: Chromatin accessibility in EVT cells. P values for called peaks estimated by a Poisson model followed by Benjamini and Hochberg procedure to adjust for multiple testing implemented in MACS3.

File Name: Supplementary Data 4a

Description: Differential binding affinity in chromatin accessibility regions in stem state cells. P values for called peaks and differential binding estimated by a Poisson model followed by Benjamini and Hochberg procedure to adjust for multiple testing implemented in MACS3 and DiffBind.

File Name: Supplementary Data 4b

Description: Differential binding affinity in chromatin accessibility regions in EVT cells. P values for called peaks and differential binding estimated by a Poisson model followed by Benjamini and Hochberg procedure to adjust for multiple testing implemented in MACS3 and DiffBind.

File Name: Supplementary Data 5

Description: Differential gene expression in EVT cells vs Stem state cells for genes mapping near (within 10kb) a chromatin accessibility region with differential binding and greater accessibility in EVT cells. P values for differential expression estimated by a Wald test followed by Benjamini and Hochberg procedure to adjust for multiple testing implemented in DESeq2. P

values for called peaks and differential binding estimated by a Poisson model followed by Benjamini and Hochberg procedure to adjust for multiple testing implemented in MACS3 and DiffBind.

File Name: Supplementary Data 6

Description: 'Super-enhancers' overlapping a chromatin accessibility region with differential binding and greater accessibility in EVT cells. P values for called peaks and differential binding estimated by a Poisson model followed by Benjamini and Hochberg procedure to adjust for multiple testing implemented in MACS3 and DiffBind. P values for super-enhancers estimated by a Poisson model followed by Benjamini and Hochberg procedure to adjust for multiple testing implemented in HOMER.

File Name: Supplementary Data 7a

Description: Chromatin loops identified by Hi-C in stem state cells

File Name: Supplementary Data 7b

Description: Chromatin loops identified by Hi-C in EVT cells

File Name: Supplementary Data 8a

Description: Genes near (10kb) chromatin loop anchors where both anchors overlap a chromatin accessibility region with differential binding and greater accessibility in EVT cells. P values for differential expression estimated by a Wald test followed by Benjamini and Hochberg procedure to adjust for multiple testing implemented in DESeq2. P values for called peaks and differential binding estimated by a Poisson model followed by Benjamini and Hochberg procedure to adjust for multiple testing implemented in MACS3 and DiffBind.

File Name: Supplementary Data 8b

Description: Genes near (10kb) chromatin loop anchors where one anchor overlaps a chromatin accessibility region with differential binding and greater accessibility in EVT cells. P values for differential expression estimated by a Wald test followed by Benjamini and Hochberg procedure to adjust for multiple testing implemented in DESeq2. P values for called peaks and differential binding estimated by a Poisson model followed by Benjamini and Hochberg procedure to adjust for multiple testing implemented in MACS3 and DiffBind.

File Name: Supplementary Data 9a

Description: TF motif enrichment in chromatin accessibility region with differential binding and greater accessibility in EVT cells. P values estimated by a binomial model implemented in HOMER.

File Name: Supplementary Data 9b

Description: TF motif enrichment in differentially bound ATAC-Seq peaks overlapping H3K27ac mark in EVT cells. P values estimated by a binomial model implemented in HOMER.

File Name: Supplementary Data 9c

Description: TF motif enrichment in differentially bound ATAC-Seq peaks in EVT cells overlapping a 'super-enhancer'. P values estimated by a binomial model implemented in HOMER.

File Name: Supplementary Data 10

Description: Differential gene expression in EVT cells vs Stem state cells for transcription factor genes with motifs top ranked across all datasets. P values for differential expression estimated by a Wald test followed by Benjamini and Hochberg procedure to adjust for multiple testing implemented in DESeq2.

File Name: Supplementary Data 11

Description: Topmost differentially expressed EVT cell genes mapping near (10kb) chromatin loop anchors where one or both anchors overlap a chromatin accessibility region with differential binding and greater accessibility in EVT cells. P values for differential expression estimated by a Wald test followed by Benjamini and Hochberg procedure to adjust for multiple testing implemented in DESeq2.

File Name: Supplementary Data 12a

Description: Pathway analysis of top 500 most differentially expressed genes in EVT cells upon SNAI1 disruption. P values calculated using a right-tailed Fisher Exact Test implemented in Ingenuity Pathway analysis software.

File Name: Supplementary Data 12b

Description: Pathway analysis of top 500 most differentially expressed genes in EVT cells upon EPAS1 disruption. P values calculated using a right-tailed Fisher Exact Test implemented in Ingenuity Pathway analysis software.

File Name: Supplementary Data 13

Description: Differentially expressed genes in EVT cells upon EPAS1 disruption mapping to Cell Migration function.

File Name: Supplementary Data 14

Description: Chromatin accessibility regions with differential binding assessment in EVT cells overlapping EPAS1 binding motif and associated expression change of nearby gene upon EPAS1 disruption. P values for called peaks and differential binding estimated by a Poisson model followed by Benjamini and Hochberg procedure to adjust for multiple testing implemented in MACS3 and DiffBind. P values for differential expression estimated by a Wald test followed by Benjamini and Hochberg procedure to adjust for multiple testing implemented in DESeq2.

File Name: Supplementary Data 15

Description: Differentially expressed genes in EVT single-cells derived from idiopathic recurrent pregnancy loss vs normal control placentas. P values for differential expression estimated by a Wald test followed by Benjamini and Hochberg procedure to adjust for multiple testing implemented in DESeq2.

File Name: Supplementary Data 16

Description: Summary statistics from GWAS results in the EPAS1 gene region. P values estimated by a linear regression model.

File Name: Supplementary Data 17

Description: Expression of genes identified in GWAS of fetal genetic variants and birth weight following EPAS1 disruption in EVT cells. P values for GWAS estimated by a linear regression model. P values for differential expression estimated by a Wald test followed by Benjamini and Hochberg procedure to adjust for multiple testing implemented in DESeq2.
