# Supplementary material for: Extravillous trophoblast cell lineage development is associated with active remodeling of the chromatin landscape
Source: Nat Commun. 2023 Aug 10;14:4826. doi: 10.1038/s41467-023-40424-5 (PMC10415281; doi:10.1038/s41467-023-40424-5)
Supplement: Supplementary file 5 — Reporting Summary [file 41467_2023_40424_MOESM5_ESM.pdf]

nature portfolio

Corresponding author(s): Kaela M. Varberg, Michael J. Soares and Elin Grundberg

Last updated by author(s): Jun 19, 2023

Reporting Summary

Nature Portfolio wishes to improve the reproducibility of the work that we publish. This form provides structure for consistency and transparency in reporting. For further information on Nature Portfolio policies, see our Editorial Policies and the Editorial Policy Checklist.

Statistics

For all statistical analyses, confirm that the following items are present in the figure legend, table legend, main text, or Methods section.

n/a	Confirmed	
☐	☒	The exact sample size (*n*) for each experimental group/condition, given as a discrete number and unit of measurement
☐	☒	A statement on whether measurements were taken from distinct samples or whether the same sample was measured repeatedly
☐	☒	The statistical test(s) used AND whether they are one- or two-sided<br>*Only common tests should be described solely by name; describe more complex techniques in the Methods section.*
☒	☐	A description of all covariates tested
☐	☒	A description of any assumptions or corrections, such as tests of normality and adjustment for multiple comparisons
☐	☒	A full description of the statistical parameters including central tendency (e.g. means) or other basic estimates (e.g. regression coefficient) AND variation (e.g. standard deviation) or associated estimates of uncertainty (e.g. confidence intervals)
☐	☒	For null hypothesis testing, the test statistic (e.g. *F*, *t*, *r*) with confidence intervals, effect sizes, degrees of freedom and *P* value noted<br>*Give P values as exact values whenever suitable.*
☒	☐	For Bayesian analysis, information on the choice of priors and Markov chain Monte Carlo settings
☒	☐	For hierarchical and complex designs, identification of the appropriate level for tests and full reporting of outcomes
☐	☒	Estimates of effect sizes (e.g. Cohen's *d*, Pearson's *r*), indicating how they were calculated

Our web collection on statistics for biologists contains articles on many of the points above.

Software and code

Policy information about availability of computer code

Data collection

No software used for data collection

Data analysis

No custom codes or software were used.

Flow cytometry data was analyzed with FlowJo v10.9.

Basic quality control of raw ATAC FASTQ files was performed using HTStream (Version 1.3.2; https://github.com/s4hts/HTStream). PhiX reads were removed using hts_SeqScreener with default parameters. Duplicates were removed using hts_SuperDeduper (-e 250000). Adapter sequences were trimmed with hts_AdapterTrimmer (-p 4). Unknown nucleotides (N) were removed using hts_Ntrimmer. Base quality trimming was performed using a minimum average quality score of 20 (-q 20) in a 10-bp sliding window (-w10) with hts_QWindowTrim. Trimmed reads shorter than 50 bp as well as orphaned reads from a pair were removed using hts_LengthFilter (-n -m 50). Reads were aligned to the human genome (GRCh38.86) using BWA mem (Version 0.7.17-r1188). The alignments were shifted using alignmentSieve (deepTools; Version 3.5.1) using the --ATACshift option. Alignments in the ENCODE blacklisted regions (https://doi.org/10.5281/zenodo.1491733) were removed using Bedtools intersect (with option `-v`; Version 2.30.0). Peaks for each sample were called independently using the callpeak function in MACS3 (Version 3.0.0a6) with a minimum FDR of 0.01 (-q 0.01).

Raw RNA-Seq FASTQ files were trimmed using default parameters (-r 0.1 -d 0.03) in Skewer (Version 0.2.2) and reads shorter than 18 bp were discarded. Transcripts were quantified using Kallisto (Version 0.46.2). Differentially expressed genes at FDR of 0.05 were discovered using the Bioconductor package DESeq2 in R (Version 1.32.0).

To discover chromatin loops the standard Juicer pipeline was used where contact maps for each sample were generated using the Arima

Genomics fragment map file specific to GRCh38 and default parameters. After merging contact maps for each cell state, stem and EVT, chromatin loops were discovered with HiCCUPS (Juicer Tools Version 1.22.01;61) using a matrix size of 1000 (-m 1000) at 5kb, 10 kb, and 25 kb resolution (-r 25000,10000,5000). Differential loops between EVT and stem were identified using HiCCUPSDiff (Juicer Tools Version 1.22.01;61) with a matrix size of 1000 (-m 1000). The chromatin loops as well as the differential loops were filtered to exclude contacts in the ENCODE blacklisted regions (https://doi.org/10.5281/zenodo.1491733) using Bedtools intersect (with option `-v`; Version 2.30.0;94). Furthermore, genes with a TSS within 10 kb of a loop region were linked to each loop.

The Cell Ranger matrix file from the single-cell RNA sequencing (scRNA-Seq) of RPL samples was processed along with the Cell Ranger matrices of two publicly available datasets of gestational age-matched normal control POC samples profiled using the same platform (10X Genomics) using standard workflow implemented in Seurat 4.1.1. Each sample was individually processed with the SCTransform function using the glmGamPoi package in Bioconductor 1.30.18 using R 4.1 with capture as a batch variable when applicable. The three datasets were integrated using the standard integration workflow for data normalized with SCTransform and 3,000 integration features were selected for downstream analysis. After integration, linear dimensional reduction, nonlinear dimensional reduction, nearest neighbor finding, and unsupervised clustering were completed according to the standard workflow. Cell type label transfer was performed by referencing the Vento-Tormo dataset. The predicted cell identities (ID) were generated using the FindTransferAnchors and TransferData functions of Seurat. If necessary, cell type labels were manually adjusted based on marker gene expression to exclude ambiguous cell clusters. Cells with greater than 50% mitochondrial reads and less than 2,000 molecules were removed from all three datasets. The Vento-Tormo dataset had an additional filtering where cells with a mitochondrial ratio of greater than 20% and less than 3500 Unique Molecular Identifiers (UMIs) and 1250 unique genes were removed. For differential expression analysis, genes were filtered according to the parameters used for Vento-Tormo dataset66. The raw UMI counts of samples with multiple captures were summed prior to differential expression analysis. Genes that were expressed in three or more cells per dataset were kept for pseudobulk differential expression analysis. A matrix was aggregated to find the UMI counts by gene per sample ID within a specific cell type. Using the raw counts and metadata of a specific cell type, a DESeq dataset was created, and the counts were transformed by the vst function for principal component analysis. Differential expression testing was performed by the DESeq function with default parameters on the generated DESeq dataset. Pairwise differential expression contrasts were performed according to the inferred cell type labels (EVT or SCT/VCT) where Vento-Tormo and Suryawanshi datasets were combined as the control group. The contrast was performed with the results function from DESeq2 using Bioconductor 1.30.18 with default parameters (Benjamini-Hochberg p-value adjustment) and alpha equal to 0.0599. Log2 fold changes were shrunk with the lfcShrink function with the apeglm package. For a gene to be significantly differentially expressed, the Benjamini-Hochberg adjusted p-value was less than 0.05.

For manuscripts utilizing custom algorithms or software that are central to the research but not yet described in published literature, software must be made available to editors and reviewers. We strongly encourage code deposition in a community repository (e.g. GitHub). See the Nature Portfolio guidelines for submitting code & software for further information.

Data

Policy information about availability of data

All manuscripts must include a data availability statement. This statement should provide the following information, where applicable:

- Accession codes, unique identifiers, or web links for publicly available datasets
- A description of any restrictions on data availability
- For clinical datasets or third party data, please ensure that the statement adheres to our policy

Source data are provided with this manuscript. Raw and processed sequencing data from ATAC-Seq, RNA-Seq, Hi-C and scRNA-Seq have been submitted to the NCBI Gene Expression Omnibus (GEO) under the following accession numbers GSE204722 (https://www.ncbi.nlm.nih.gov/geo/query/acc.cgi?acc=GSE204722) and GSE204723 (https://www.ncbi.nlm.nih.gov/geo/query/acc.cgi?acc=GSE204723). The ChIP-seq data of histone modifications are deposited in Japanese Genotype-phenotype Archive (JGA) under the accession number JGAS000107 (https://humandbs.biosciencedbc.jp/en/hum0086-v3) and JGAS000112 (https://humandbs.biosciencedbc.jp/en/hum0112-v1). Raw and processed data are available under unrestricted access.
Processed data can be visualized in the UCSC Genome Browser using the following link: https://genome.ucsc.edu/s/cmri_gmc_bioinformatics/EVT_STEM_Epigenome_Review
Additional scRNA-Seq data sets used in the study are available in ArrayExpress under the accession number E-MTAB-6701 (https://www.ebi.ac.uk/biostudies/arrayexpress/studies/E-MTAB-6701) and in BioProject under the accession number PRJNA492324 (https://www.ncbi.nlm.nih.gov/bioproject/?term=PRJNA492324).
Summary statistics from UK Biobank GWAS was accessed at http://www.nealelab.is/uk-biobank.
The reference genome GRCh38 (GCA_000001405.22) used in this study is available at http://ftp.ensembl.org/pub/release-86/fasta/homo_sapiens/dna/Homo_sapiens.GRCh38.dna.primary_assembly.fa.gz

Human research participants

Policy information about studies involving human research participants and Sex and Gender in Research.

Reporting on sex and gender	Sex is reported for both cell lines. We focus all analysis on autosomes thus the findings are applicable to both sexes and no sex-specific analysis was performed.
Population characteristics	Study participants were screened at the University of Kansas Health System Advanced Reproductive Medicine Clinic and nominated for participation by the provider based on inclusion criteria listed below. Participants did not self-nominate for participation thus no selection biases were present.<br><br>Inclusion criteria was age >18 years and <42 years at the time of conception, BMI >18 kg/m2 and <30 kg/m2, and a history of unexplained recurrent pregnancy loss (RPL) with recent diagnosis of miscarriage. Diagnosis of RPL was made using recommendations by the American Society for Reproductive Medicine Practice Committee with RPL defined as "spontaneous loss of two or more pregnancies." For all patients known etiologies for miscarriage were ruled out before allowing participation in study including: Mullerian anomaly, polycystic ovary disease, thyroid disease, diabetes, coagulopathy,

	balanced translocation or structural rearrangement from sperm or oocyte contributor, and obesity. Miscarriage was diagnosed using American College of Obstetrician and Gynecologists Committee on Practice, which defines miscarriage as "a nonviable, intrauterine pregnancy with either an empty gestational sac or a gestational sac containing an embryo or fetus without fetal cardiac activity within the first 12 6/7 weeks of gestation." All study participants had transvaginal ultrasound to confirm diagnosis of miscarriage. Product of conception (POC) including placental and decidual tissue were collected from the study participants where all samples had normal karyotype based on clinical cytogenetics testing including chromosomal analysis (GTG banded chromosomes analyzed at the 450-550 band levels) and array comparative genomic hybridization performed in accordance with current International Standing Committee on Human Cytogenetic Nomenclature (ISCN 2009)
Recruitment	See population characteristics section
Ethics oversight	This study using deidentified TS cells obtained by Drs. Okae and Arima complies with all relevant ethical regulations as approved by the Human Research Protection Program and the Human Stem Cell Research Oversite Committee at the University of Kansas Medical Center who determined the study as non-human subjects' research. Prior approval for use of human placental tissue specimens was granted by the respective local human research ethics review committees at the Mount Sinai Hospital and the University of Kansas Medical Center. The RPL study was approved by the Children's Mercy Institutional Review Board (Study No. 11120514). Informed written consent was obtained from the RPL participants before study inclusion. Participants were not compensated for study participation

Note that full information on the approval of the study protocol must also be provided in the manuscript.

Field-specific reporting

Please select the one below that is the best fit for your research. If you are not sure, read the appropriate sections before making your selection.

☒ Life sciences ☐ Behavioural & social sciences ☐ Ecological, evolutionary & environmental sciences

For a reference copy of the document with all sections, see nature.com/documents/nr-reporting-summary-flat.pdf

Life sciences study design

All studies must disclose on these points even when the disclosure is negative.

Sample size	No statistical method were used to predetermine the sample size. The sample size (2 independent donor lines) was deemed sufficient for the characterization of the global transcriptome and epigenome landscape in human trophoblasts and their transition into extravillous trophoblast cells and for the identification of key transcription regulators.
Data exclusions	No data sets derived from the human TS model were excluded from any analyses. Prior to integration of publicly available scRNA data sets from 1st trimester placental samples, three data sets were excluded from the Vento-Tormo study as they did not match (based on gestational age etc) the RPL study group. Following data integration, one sample was excluded from the Suryawanshi study due to poor quality with no cells retained after standard quality filtering procedures.
Replication	Experiments utilizing the TS model were independently repeated a minimum of three times for all results from experiments displayed as representative data (e.g., immunolocalization and immunocytochemistry images).<br>In addition, to verify the reproducibility of the findings the following was performed where results were successfully validated:<br>1) Orthogonal validation in additional donor lines for all NGS and gene-based analysis including gene silencing experiments.<br>2) Orthogonal validation of in vitro results (TS model) using in vivo single-cell RNA expression data sets<br>3) Validation of RNA-Seq results by RT-PCR and protein analysis by Western blot<br>4) Validation of ATAC-Seq results by histone modification ChIP-Seq<br>5) Independent replication of GWAS results using UK Biobank
Randomization	No randomizations were done. Covariates such as gestational age and fetal diagnosis was taken into account in scRNA-Seq analysis to match the cases with RPL.
Blinding	Cause of pregnancy loss was blinded during data integration and initial analysis that included filtering and clustering of the scRNA-Seq data.

Reporting for specific materials, systems and methods

We require information from authors about some types of materials, experimental systems and methods used in many studies. Here, indicate whether each material, system or method listed is relevant to your study. If you are not sure if a list item applies to your research, read the appropriate section before selecting a response.

Materials & experimental systems

n/a	Involved in the study
☐	☒ Antibodies
☐	☒ Eukaryotic cell lines
☒	☐ Palaeontology and archaeology
☒	☐ Animals and other organisms
☒	☐ Clinical data
☒	☐ Dual use research of concern

Methods

n/a	Involved in the study
☐	☒ ChIP-seq
☐	☒ Flow cytometry
☒	☐ MRI-based neuroimaging

Antibodies

Antibodies used

All antibody information including dilution, supplier name, catalog number, clone name, and lob numbers (when applicable) are included in the respective methods sections of the manuscript. These details are also outlined below:
H3K4me3 (1:500, Clone No. CMA304, MABI0304, MBL International, Woburn, MA)
H3K27ac (1:150, MABI0309, Clone No. CMA309, MBL International, Woburn, MA)
AP-2 gamma (1:50, 2320, Cell Signaling Technology, anti-rabbit)
SNAI1 (1:1000, 3879S, Cell Signaling Technology)
EPAS1 (1:1000, 66731-1-Ig, Proteintech)
TFAP2C (1:750, 6E4/4, sc-12762, Santa Cruz Biotechnology)
Glyeraldehyde-3-phosphate dehydrogenase (GAPDH, 1:5000, AM4300, Thermo-Fisher Scientific)
Secondary antibody: goat anti-rabbit IgG HRP, 1:5000, 7074S, Cell Signaling Technology
Secondary antibody: horse anti-mouse IgG HRP, 1:5000, 7076, Cell Signaling Technology
anti-HLA-G-phycoerythin (1:1500, 1P-292-C100, Cedarlane Labs)

Validation

SNAI1, EPAS1, and TFAP2C antibodies were validated using loss-of-function strategies in human trophoblast stem cells. shRNA knockdown experiments targeting SNAI1, EPAS1, or TFAP2C were performed in two biologic replicates (two different TS cell lines) a minimum of three times per cell line. shRNA depletion was validated by western blot using the aforementioned antibodies specific to these targets. Band sizes were apparent at appropriate molecular weights and selected depletion of all gene targets evident in shRNA group compared to control. This data is presented in the manuscript in Figures 6, 8, 9 and Supplementary Figures 19, 22, and 23. GAPDH and all secondary antibodies have been validated in previous publications from the Soares lab.

Relevant Citations for all antibodies:
1. H3K4me3 (MABI0304, Clone No. CMA304, MBL International, Woburn, MA)
Journal: Cell Struct Funct.
Title: The organization of histone H3 modifications as revealed by a panel of specific monoclonal antibodies.
Author: Kimura H, et. al.
Year: 2008

2. H3K27ac (MABI0309, Clone No. CMA309, MBL International, Woburn, MA)
Journal: Cell Struct Funct.
Title: The organization of histone H3 modifications as revealed by a panel of specific monoclonal antibodies.
Author: Kimura H, et. al.
Year: 2008

3. AP-2 gamma (2320, Cell Signaling Technology, anti-rabbit):
Journal: Cell Discov.
Title: Generation of human blastocyst-like structures from pluripotent stem cells.
Author: Yong Fan, et. al.
Year: 2021

4. SNAI1 (1:1000, 3879S, Cell Signaling Technology)
Journal: Nat Commun.
Title: Non-canonical functions of SNAIL drive context-specific cancer progression.
Author: Mariel C Paul, et. al.
Year: 2023

5. EPAS1 (1:1000, 66731-1-Ig, Proteintech)
Journal: Cell Mol Gastroenterol Hepatol
Title: A Mitochondrial DNA Variant Elevates the Risk of Gallstone Disease by Altering Mitochondrial Function.
Author: Dayan Sun
Year: 2021

6. TFAP2C (1:750, 6E4/4, sc-12762, Santa Cruz Biotechnology)
Journal: Neoplasia
Title: AP-2γ Induces p21 Expression, Arrests Cell Cycle, and Inhibits the Tumor Growth of Human Carcinoma Cells1
Author: Hualei Li
Year: 2006

7. Glyeraldehyde-3-phosphate dehydrogenase (GAPDH, 1:5000, AM4300, Thermo-Fisher Scientific)
Journal: Blood Adv.

Title: MiR-146b-5p regulates IL-23 receptor complex expression in chronic lymphocytic leukemia cells
Author: Serena Matis.
Year: 2022

8. Secondary antibody: goat anti-rabbit IgG HRP, 1:5000, 7074S, Cell Signaling Technology
Journal: Nature Communications
Title: Endothelial FAT1 inhibits angiogenesis by controlling YAP/TAZ protein degradation via E3 ligase MIB2
Author: Rui Li
Year: 2023

9. Secondary antibody: horse anti-mouse IgG HRP, 1:5000, 7076, Cell Signaling Technology
Journal: Nature Communications
Title: Endothelial FAT1 inhibits angiogenesis by controlling YAP/TAZ protein degradation via E3 ligase MIB2
Author: Rui Li
Year: 2023

10. anti-HLA-G-phycoerythin (1:1500, 1P-292-C100, Cedarlane Labs, Exbio)
Journal: Journal of Autoimmunity
Title: Tolerogenic IL-10-engineered dendritic cell-based therapy to restore antigen-specific tolerance in T cell mediated diseases
Author: Laura Passeri
Year: 2023

Eukaryotic cell lines

Policy information about cell lines and Sex and Gender in Research

Cell line source(s): Cell lines were obtained from Okae et al. at Tohoku University in Japan through a MTA. CT27 (female) and CT29 (male) human trophoblast stem cell lines were utilized. These lines had previously been established prior to this work. These human trophoblast stem cell lines are also now available commercially through the Riken Cell Bank https://cell.brc.riken.jp/en/

Authentication: Cell lines were karyotyped for authentication of sex, etc.

Mycoplasma contamination: Cell lines had been confirmed negative for mycoplasma by Okae et al. prior to distribution.

Commonly misidentified lines (See ICLAC register): No commonly misidentified cell lines were used in the study.

ChIP-seq

Data deposition

☒ Confirm that both raw and final processed data have been deposited in a public database such as GEO.

☒ Confirm that you have deposited or provided access to graph files (e.g. BED files) for the called peaks.

Data access links
May remain private before publication.
https://www.ncbi.nlm.nih.gov/geo/query/acc.cgi?acc=GSE204722

Files in database submission:

ChIP_TS-CT1-EVT-K27ac_R1.fastq.gz
ChIP_TS-CT1-EVT-K27ac_R2.fastq.gz
ChIP_TS-CT1-EVT-K4me3_R1.fastq.gz
ChIP_TS-CT1-EVT-K4me3_R2.fastq.gz
ChIP_TS-CT1-Input_R1.fastq.gz
ChIP_TS-CT1-Input_R2.fastq.gz
ChIP_TS-CT1-K27ac_R1.fastq.gz
ChIP_TS-CT1-K27ac_R2.fastq.gz
ChIP_TS-CT1-K4me3_R1.fastq.gz
ChIP_TS-CT1-K4me3_R2.fastq.gz

ChIP_TS-CT1-K27ac_peaks.narrowPeak
ChIP_TS-CT1-EVT-K27ac_peaks.narrowPeak
ChIP_TS-CT1-EVT-K4me3_peaks.narrowPeak
ChIP_TS-CT1-K4me3_peaks.narrowPeak

ChiP_CT27_Stem_1_S28_L003_R1_001.fastq.gz
ChiP_CT27_Stem_1_S28_L003_R2_001.fastq.gz
ChiP_CT27_Stem_2_S29_L003_R1_001.fastq.gz
ChiP_CT27_Stem_2_S29_L003_R2_001.fastq.gz
ChiP_CT27_Stem_3_S30_L003_R1_001.fastq.gz
ChiP_CT27_Stem_3_S30_L003_R2_001.fastq.gz
ChiP_CT27_Stem_Input_1_S31_L003_R1_001.fastq.gz
ChiP_CT27_Stem_Input_1_S31_L003_R2_001.fastq.gz
ChiP_CT27_Stem_Input_2_S32_L003_R1_001.fastq.gz

ChiP_CT27_Stem_Input_2_S32_L003_R2_001.fastq.gz
ChiP_CT27_Stem_Input_3_S33_L003_R1_001.fastq.gz
ChiP_CT27_Stem_Input_3_S33_L003_R2_001.fastq.gz

ChiP_CT27_Stem_2_peaks.narrowPeak
ChiP_CT27_Stem_1_peaks.narrowPeak
ChiP_CT27_Stem_3_peaks.narrowPeak

Genome browser session (e.g. UCSC)

https://genome.ucsc.edu/s/cmri_gmc_bioinformatics/EVT_STEM_Epigenome_Review

Methodology

Replicates

One technical replicate was used for each histone ChIP including input for each antibody used. For TFAP2C, three biological replicates were used each with two technical replicates.

Sequencing depth

Samples were sequenced to an average depth of 20M paired end reads with 100bp read length

Antibodies

H3K4me3 (Clone No. CMA304, MABI0304, MBL International, Woburn, MA)
H3K27ac (MABI0309, Clone No. CMA309, MBL International, Woburn, MA)
TFAP2C (1:750, 6E4/4, sc-12762, Santa Cruz Biotechnology)

Peak calling parameters

Peaks for each sample were called independently using the callpeak function in MACS3 (Version 3.0.0a6) with a minimum FDR of 0.01 (-q 0.01).

Data quality

68758 and 80013 H3K27Ac peaks were identified in EVT cells and stem state cells respectively.
21946 and 24325 H3K4me3 peaks were identified in EVT cells and stem state cells respectively.
19657, 24341 and 41766 TFAP2C peaks were identified in the three replicates of which 15502 was seen across all three ChIP and used as high-quality set in analysis.
Quality was ensured by overlapping peaks identified by ATAC-Seq in the same cell line
The FRiP (fraction of reads in peaks) was between 37-74 across the four datasets

Software

No additional software was used to analyze the data

Flow Cytometry

Plots

Confirm that:

☒ The axis labels state the marker and fluorochrome used (e.g. CD4-FITC).

☒ The axis scales are clearly visible. Include numbers along axes only for bottom left plot of group (a 'group' is an analysis of identical markers).

☒ All plots are contour plots with outliers or pseudocolor plots.

☒ A numerical value for number of cells or percentage (with statistics) is provided.

Methodology

Sample preparation

Cells in culture were washed with phosphate buffered saline (PBS), detached with TrypLE Express (12604021, Thermo-Fisher Scientific), and collected in basal culture medium. Cell suspensions were centrifuged, cell pellets were washed with PBS, and resuspended with 4% paraformaldehyde in PBS for 20 min at room temperature with gentle agitation. Fixed cell suspensions were centrifuged, and cell pellets were washed twice with PBS and stored at 4°C. Fixed cells were permeabilized with PBS containing 3% BSA and 0.2% Triton X-100 for 30 min at room temperature with gentle agitation. Cells were washed with PBS and blocked with PBS containing 3% BSA for 15 min and then incubated overnight with anti-HLA-G-phycoerythin (1:1500, 1P-292-C100, Cedarlane Labs) prepared in PBS containing 3% BSA and 0.2% Triton X-100 in the dark at 4°C with gentle agitation. Cells were washed twice with PBS, filtered, and analyzed using a BD LSR II flow cytometer (BD Biosciences) at the University of Kansas Medical Center (KUMC) Flow Cytometry Core Laboratory.

Instrument

BD LSR II (BD Biosciences)

Software

FlowJo v10.9

Cell population abundance

Relevant population abundance: majority

Gating strategy

Initial gates were used to select for cells (discard debris, etc.) (Supplemental Figure 2A) secondary gates were used to select for single cells to discard doublets/cells sticking together (Supplemental Figure 2B). Finally, the third set of gates were used to discern PE+/- cells (Supplemental Figure 2C).

☒ Tick this box to confirm that a figure exemplifying the gating strategy is provided in the Supplementary Information.
